# AE-MXene-modified titanium alloy promotes osseointegration by regulating the AMPK-MTOR-autophagy pathway in macrophage

**DOI:** 10.1186/s12951-026-04080-3

**Published:** 2026-02-03

**Authors:** Rui Chao, Lei Sun, Xinyu Xu, Zhan Liu, Xinyi Xu, Zhen Ren, Xinwei Chen, Weifeng Xu, Xuzhuo Chen, Ying Hu, Shanyong Zhang

**Affiliations:** 1 Department of Oral Surgery, Shanghai Ninth People’s Hospital, Shanghai Jiao Tong University School of Medicine; , College of Stomatology, Shanghai Jiao Tong University; National Center for Stomatology; National Clinical Research Center for Oral Diseases; Shanghai Key Laboratory of Stomatology; Shanghai Research Institute of Stomatology, 200011 Shanghai, China; 2 Hefei Stomatology Hospital and Hefei Clinical School of Stomatology, Anhui Medical University, Hefei, 230001 Anhui Province China; 3 Anhui Province Key Lab of Aerospace Structural Parts Forming Technology and Equipment, School of Materials Science and Engineering, Hefei University of Technology, Hefei, 230009 China

**Keywords:** Titanium, Surface modification, MXene, Macrophage, Osseointegration

## Abstract

Effective osseointegration requires successful interaction between an implant and the local bone and immune environments. Surface modification presents a promising strategy to enhance the biocompatibility and integration of titanium implants. Although emerging research on transition metal carbides and nitrides (MXenes) demonstrates their potential to improve implant integration by modulating macrophage behavior and osteogenesis, existing studies have not explored synergistic modification strategies or the specific molecular mechanisms linking immunomodulation to bone healing. To address this, we developed a novel alkali-etched MXene (AE-MXene) coating by integrating alkali etching with MXene nanosheet loading, creating a platform that simultaneously optimizes micro/nanoscale surface topography and bioactive functionality—a synergistic approach previously unreported for MXene-based implants. Through comprehensive in vitro and in vivo analyses, we demonstrate that the AE-MXene surface possesses potent antibacterial, anti-inflammatory, and pro-osteogenic properties. Notably, we reveal for the first time that AE-MXene activates the AMP-activated protein kinase (AMPK)–mechanistic target of rapamycin (mTOR) pathway in macrophages, significantly upregulating autophagy to drive enhanced osteogenesis and angiogenesis. These findings delineate a unique autophagy-mediated mechanism through which AE-MXene promotes osseointegration, distinguishing it from prior MXene implant studies and highlighting its therapeutic potential for immunomodulatory and antimicrobial applications.

**Graphical abstract:**

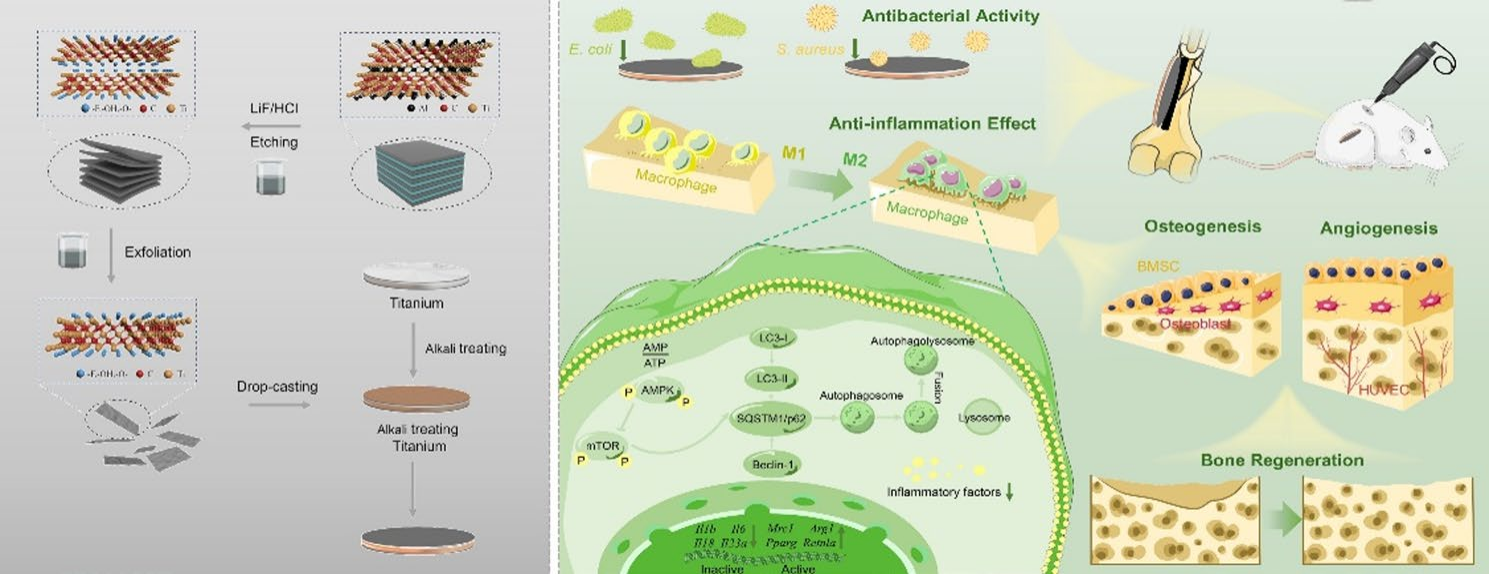

**Supplementary Information:**

The online version contains supplementary material available at 10.1186/s12951-026-04080-3.

## Background

Joint replacement surgery, which utilizes prostheses to replace damaged bone, represents a primary treatment for end-stage osteoarthropathies [1, 2]. Titanium and its alloys are extensively employed in orthopedic [3, 4] and dental [5] implants owing to their excellent biocompatibility and mechanical strength. However, their inherent biological inertness [6, 7] and susceptibility to bacterial colonization [8, 9] can result in poor osseointegration, delayed healing, and eventual implant failure.

To address these limitations, various surface modification strategies have been developed. The spatial [10] and physical [11, 12] design of an implant surface can modulate immune responses by influencing macrophage morphology and behavior. For instance, micro-rough titanium surfaces with superhydrophilic properties have been shown to promote macrophage polarization toward the anti-inflammatory M2 phenotype, facilitating the secretion of osteogenic growth factors that support osteoblast differentiation [13, 14]. Among these strategies, alkaline etching (AE) is notable for creating superhydrophilic, nanoscale rough surfaces that mimic natural bone architecture and can promote anti-inflammatory macrophage polarization [15–17]. Nonetheless, surface topography alone is often insufficient to fully combat bacterial infection and chronic inflammation.

In recent years, two-dimensional transition metal carbides and nitrides (MXenes) have emerged as promising coatings for biomedical implants [18–20]. A growing body of research has established that MXene-based materials can directly regulate the immune microenvironment to foster bone repair. For example, a recent landmark study developed a scalable, ultrastrong MXene film that significantly enhances bone regeneration by efficiently clearing reactive oxygen species (ROS) and promoting macrophage polarization toward the anti-inflammatory M2 phenotype [21]. Other advanced strategies utilize external stimuli; a 2025 study demonstrated that MXene nanosheets, under the spatiotemporal control of a rotating magnetic field, can sequentially guide macrophage polarization from M1 to M2 states to heal infected wound [22]. These studies collectively underscore that modulating macrophage behavior is a key factor through which MXene coatings enhance osteogenesis.

While these independent lines of inquiry into MXene coatings or externally stimulated responses are illuminating, they predominantly focus on phenotypic outcomes, such as M2 polarization, or require external energy fields. A significant knowledge gap persists concerning the specific intracellular signaling pathways that are autonomously activated by the intrinsic properties of an implant surface to coordinate immune-mediated bone repair. Furthermore, the potential synergy between the microscale topographic cues of a modified titanium substrate and the nanoscale bioactivity of an MXene coating remains unexplored.

To address this, our work introduces a distinct strategy and an unreported mechanistic insight. We propose a novel dual-modification approach that synergistically combines alkali etching (AE) with MXene nanosheet loading to create an integrated AE-MXene platform. This strategy provides dual optimization of micro/nanoscale topography and surface bioactivity. More critically, we reveal a unique intracellular mechanism: the AE-MXene surface significantly upregulates macrophage autophagy by activating the AMPK–mTOR pathway. This autophagic response, initiated autonomously by the engineered implant surface, acts as a crucial upstream event that subsequently enhances osteogenesis and angiogenesis. These findings address a critical literature gap, providing a novel mechanistic foundation for the rational design of biomaterials to direct the bone-healing process.

## Results and discussion

### Design, synthesis, and characterization of AE-MXene

**Fig. 1:**
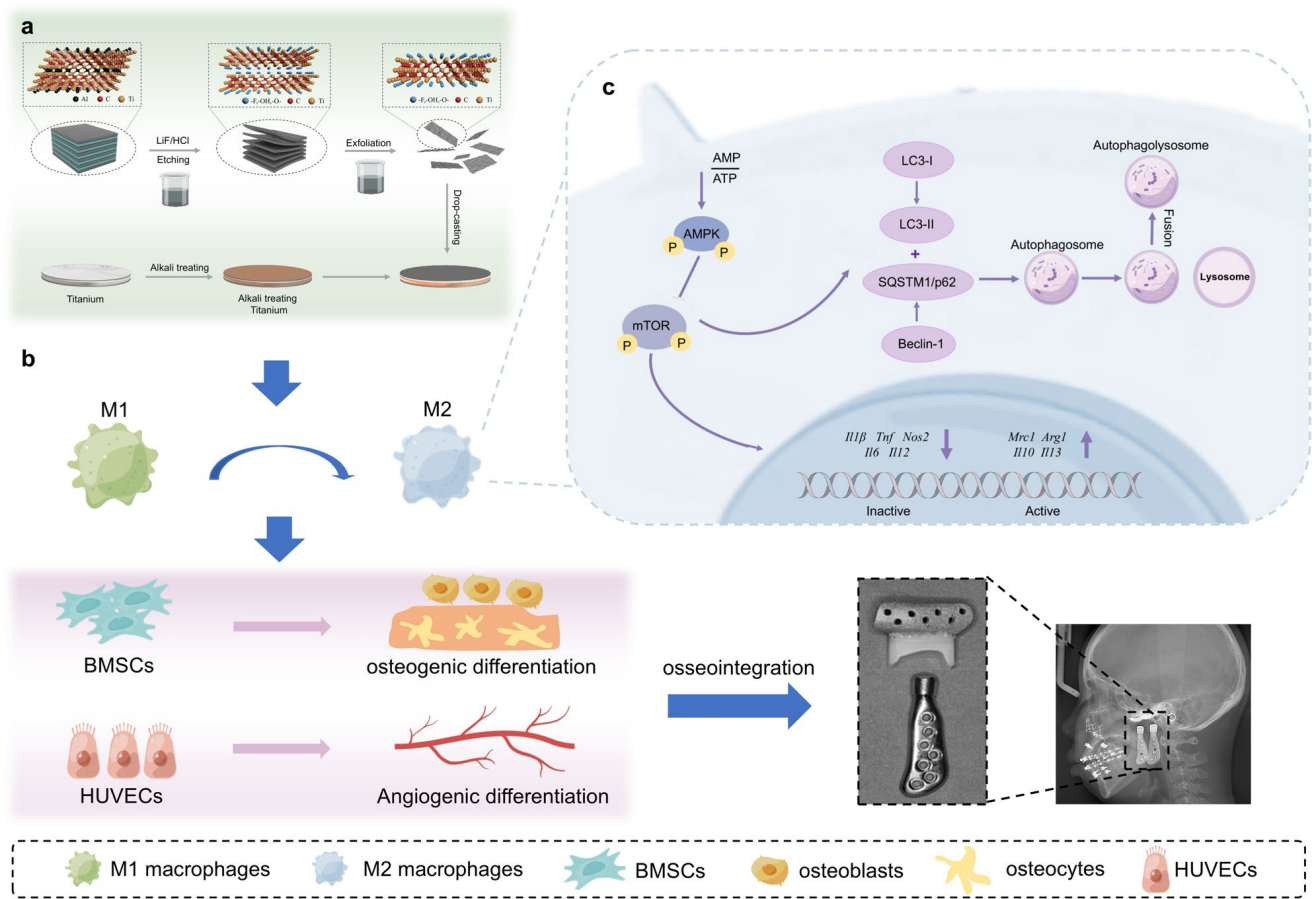
Schematic illustration of the synthesis and application of AE-MXene for multifunctional improvement of titanium prothesis. **a**) Schematic for the AE-MXene preparation. **b**) The multi-biological functions of promoting M2 polarization of macrophages, promoting osteogenesis, promoting angiogenesis, and ultimately promoting osseointegration. **c**) Schematic representation of the potential application of AE-MXene in promoting bone integration

AE-MXene coatings were fabricated via a stepwise process (Fig. 1a): Ti₃AlC₂ was etched with LiF/HCl, centrifuged to neutrality (pH 6–7) for effective interlayer exfoliation, and ultrasonicated for dispersion. Alkali-etched titanium (Ti) sheets were Drop-cast with MXene dispersion to form uniform coatings.

**Fig. 2:**
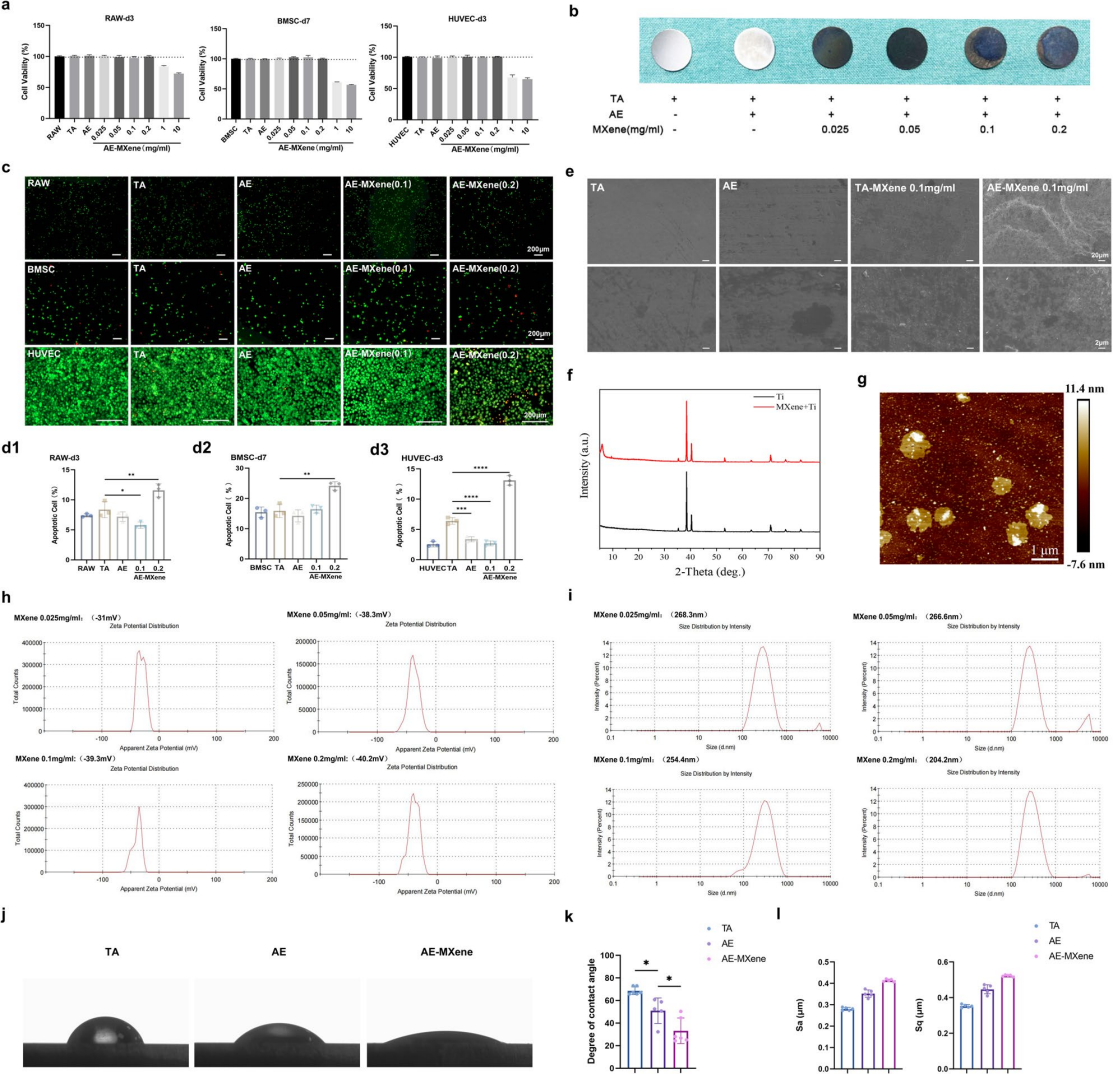
Biocompatibility and material characterization of different modified surfaces. **a**) CCK-8 assays of different MXene concentrations in BMSCs, HUVECs, and RAW264.7 cells (*n* ≥ 3). **b**) Images of titanium sheets loaded with different MXene concentrations under ordinary light sources. **c**) Live/dead staining images of CTL, TA, AE, AE-MXene (0.1 mg/mL), and AE-MXene (0.2 mg/mL). Scale bar = 200 μm. (**d**1-**d**3) Statistical plot of live/dead staining (*n* ≥ 3). **e**) Surface SEM images of TA, AE, TA-MXene (0.1 mg/mL), and AE-MXene (0.1 mg/mL). Scale bar = 20 μm (first line); 2 μm (second line). **f**) XRD patterns of Ti and MXene-coated Ti. **g**) AFM images of AE-MXene (0.1 mg/mL) surface. **h**) Zeta potential measurements of MXene dispersions at concentrations of 0.025, 0.05, 0.1, and 0.2 mg/mL. **i**) DLS analysis of the MXene dispersions at different concentrations. **j**) The water contact angle of TA, AE, and AE-MXene. **k**) The water contact angle analysis (*n* ≥ 3). **l**) Statistical plot of surface roughness (*n* ≥ 3). *P < 0.05, **P < 0.01, ***P < 0.001, and ****P < 0.0001

Biocompatibility, a prerequisite for biomedical materials, was optimized by evaluating alkali etching duration and MXene concentration. A 2-h alkali etching period was determined to be optimal for promoting the proliferation of bone marrow-derived mesenchymal stem cells (BMSCs), human umbilical vein endothelial cells (HUVECs), and RAW264.7 cells (Figure S1). Cell Counting Kit-8 (CCK-8) confirmed the non-cytotoxicity of MXene at concentrations ranging from 0.025 to 0.2 mg/mL (Fig. 2a). As shown in Fig. 2b, titanium sheets loaded with different MXene concentrations exhibited a characteristic blue-purple luster. Live/dead staining revealed minimal cell death in the AE-MXene (0.1 mg/mL) group, in contrast to increased cell mortality observed at the 0.2 mg/mL concentration (Fig. 2c and d). These findings are consistent with previous research emphasizing the concentration-dependent biocompatibility of MXene-based materials [23], but extend this insight by establishing a link between alkali etching pretreatment and improved cell viability.

Scanning electron microscopy (SEM) observations demonstrated that alkali etching enhanced Ti surface roughness, facilitating the uniform and stable deposition of MXene (Fig. 2e)—in contrast to the uneven coating formation on unetched Ti (TA-MXene group). X-ray diffraction (XRD) analysis confirmed the presence of MXene’s characteristic (002) diffraction peak at ~6.8° (Fig. 2f), while X-ray photoelectron spectroscopy (XPS) detected a prominent F 1 s peak (~683.7 eV), verifying successful MXene immobilization via fluorine-containing functional groups (Figure S2). Atomic force microscopy (AFM) analysis of AE-MXene (0.1 mg/mL) revealed a uniform surface with an average height distribution of 5–6 nm (Fig. 2g). Zeta potential and dynamic light scattering (DLS) analyses validated the colloidal stability of MXene dispersions (0.025–0.2 mg/mL): inherent electronegativity increased with concentration, while average particle size decreased (Fig. 2h and i). This inverse relationship between concentration and particle size—driven by enhanced surface charge and specific surface area—improved dispersion stability, consistent with colloid science principles [24]. Notably, this stability outperforms the MXene/HAp composite dispersions reported in a 2024 study, where aggregation was observed at concentrations > 0.1 mg/mL [25]. The superior stability of AE-MXene dispersions facilitates uniform coating deposition, a critical advantage for implant applications.

Surface hydrophilicity, a critical mediator of osseointegration [26], was significantly improved by AE-MXene modification: contact angles decreased from 68.65 ± 3.43° (TA group) to 33.2 ± 11.3° (AE-MXene group) (Fig. 2j and k). Given that enhanced hydrophilicity is well-documented to promote osteogenic differentiation and facilitate early-stage osseointegration [27], the improved wettability observed in the AE-MXene group suggests that the modified surface may exhibit superior osseointegration potential. Quantitative roughness metrics (Sa, Sq, Sz) confirmed that alkali etching significantly increased surface roughness compared to TA, and MXene modification further amplified this effect (Fig. 2l, Figure S3). The reduced surface texture isotropy (Stz) of AE-MXene is particularly notable, as anisotropic topographies have been shown to enhance osteoblast adhesion and differentiation [28]. Collectively, these characterization results confirm that AE-MXene exhibits favorable surface properties (stability, roughness, hydrophilicity) and biocompatibility, laying a solid foundation for its subsequent biological performance evaluation.

### Evaluation of anti-inflammatory, osteogenic, and angiogenic effects of AE-MXene *in vitro*

#### Immunomodulatory effects on macrophages

**Fig. 3:**
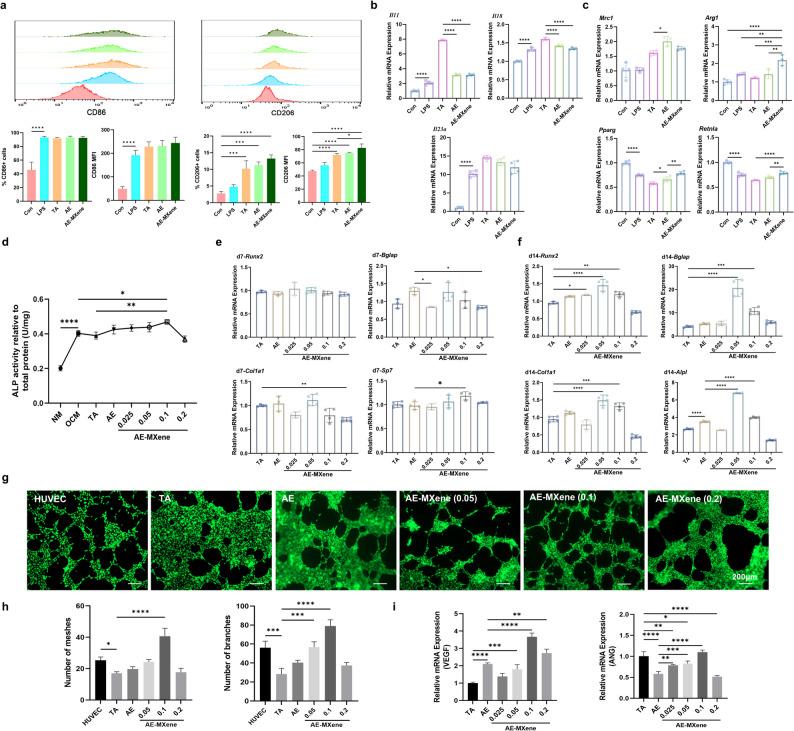
Evaluation of anti-inflammatory, osteogenesis, and angiogenesis effects of AE-MXene in vitro. **a**) Flowcytometry analysis of macrophages in the different groups. CD86 and CD206 were used as the markers for M1 and M2 phenotypes, respectively (*n* ≥ 3). **b**) Expression of M1-related genes analyzed by RT-qPCR in macrophages in the different groups (*n* ≥ 3). **c**) Expression of M2-related genes analyzed by RT-qPCR in macrophages in the different groups (*n* ≥ 3). **d**) ALP activity in different material groups after 7 days of induction culture of BMSCs (*n* ≥ 3). (**e**, **f**) Expression of osteogenesis-related genes by RT-qPCR in BMSCs in the different groups with the conditional culture on the 7th and 14th days (*n* ≥ 3). **g**) Images of HUVECs tubule formation after different material treatments for 8 h, and then stained with Calcein-AM. Scale bar = 200 μm. **h**) Quantitative analysis of the number of meshes and branches in immunofluorescence images (*n* ≥ 3). **i**) Expression of VEGF and ANG analyzed by RT-qPCR in HUVECs in the different groups with the conditional culture (*n* ≥ 3). *P < 0.05, **P < 0.01, ***P < 0.001, and ****P < 0.0001

Macrophage polarization is critical for mitigating peri-implant inflammatory osteolysis [29]. AE-MXene (0.1 mg/mL) significantly suppressed mRNA expression of pro-inflammatory cytokines (Il1b, Il6, Il23a, Il11) in RAW264.7 cells, while the 0.2 mg/mL concentration selectively inhibited Il6 and Il23a (Figure S4). Under lipopolysaccharide (LPS)-induced inflammatory conditions, flow cytometry demonstrated that AE-MXene increased CD206⁺ (M2) macrophage percentage from 4.79 ± 0.65% (LPS group) to 13.23 ± 1.11% and elevated CD206 mean fluorescence intensity (MFI) from 56.33 ± 4.31 to 82.8 ± 5.9 (Fig. 3a). RT-qPCR further confirmed downregulation of M1 markers (Il11, Il18, Il23a) and upregulation of M2 markers (Mrc1, Arg1, Pparg, Retnla) (Fig. 3b and c).

Notably, the capacity of AE-MXene to promote M2 macrophage polarization and shape a pro-regenerative immune microenvironment aligns with the consensus emerging from recent research on MXene-based biomaterials. This mechanism is widely recognized as a key driver for mitigating peri-implant inflammation and enhancing bone regeneration. Recent advances in the field further corroborate such regulatory roles of MXene: for instance, a 2025 study on Ta₄C₃ MXene demonstrated that it can promote M1-to-M2 macrophage polarization by scavenging reactive oxygen species (ROS), thereby suppressing inflammation in metabolic liver disease [30]. Collectively, AE-MXene actively modulates macrophage polarization toward the M2 phenotype to construct a pro-regenerative immune microenvironment—a well-validated key mechanism for alleviating peri-implant inflammation and supporting bone regeneration, which is consistent with the latest advances in MXene biomaterials research.

#### Osteogenic differentiation of BMSCs

Alkaline phosphatase (ALP) activity assays confirmed enhanced osteogenic potential in the AE-MXene (0.1 mg/mL) group (Fig. 3d). Furthermore, RT-qPCR analysis revealed dynamic regulation of key osteogenic genes: early upregulation (on Day 3) of Runx2, Bglap, Col1a1, and Sp7 across all AE-MXene groups (Figure S5), sustained elevation of Sp7 in the 0.1 mg/mL group on Day 7; and significant upregulation of all tested markers (Runx2, Bglap, Col1a1, Alpl) in the 0.05–0.1 mg/mL concentration range on Day 14 (Fig. 3e and f). This time-dependent osteogenic response underscores AE-MXene’s ability to sustain long-term osteogenic differentiation.

#### Angiogenic potential of HUVECs

Angiogenesis is essential for nutrient supply during osseointegration [31]. Tubule formation assays showed that AE-MXene (0.1 mg/mL) significantly increased mesh and branch numbers compared to TA (Fig. 3g and h), with a decline at 0.2 mg/mL indicating concentration-dependent effects. Vascular endothelial growth factor (VEGF) and angiopoietin (ANG) are the key regulators of angiogenesis and vascular remodeling [32]. RT-qPCR confirmed upregulation of VEGF—a fourfold increase in the 0.1 mg/mL group—and reversal of alkali-induced inhibition of ANG (Fig. 3i).

Wan et al. developed a scalable and super-strong MXene film that significantly promoted the adhesion, proliferation, and osteogenic differentiation of BMSCs and enhanced bone regeneration in rat cranial defect models; however, this study focused solely on osteogenic performance without investigating its angiogenic effects [33]. These results from the present study demonstrate the unique dual capability of AE-MXene to simultaneously promote osteogenesis and angiogenesis, thereby exhibiting great potential for optimizing osseointegration.

### AE-MXene induces autophagy in macrophages

**Fig. 4:**
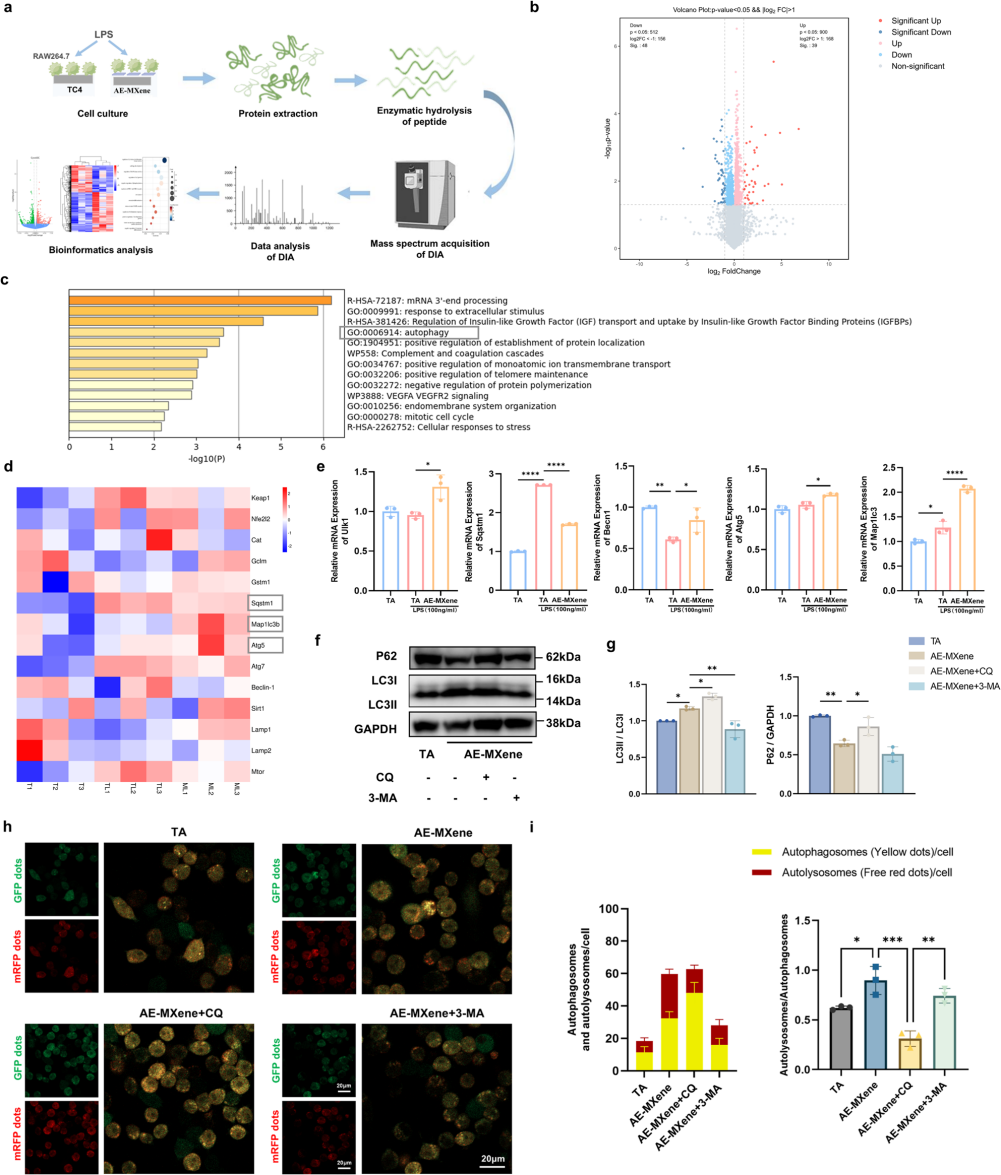
AE-MXene induced autophagy in macrophages. **a**) The basic technical flowchart of Data-Independent Acquisition (DIA)-based quantitative proteomics. **b**) Volcano plot of the distinct upregulated and downregulated genes of ML (AE-MXene (0.1 mg/mL) + LPS) versus TL (TA + LPS). **c**) Functional enrichment map of differentially expressed proteins in the TL and ML groups. **d**) Heatmap of differentially expressed genes associated with autophagy and antioxidative processes of ML versus TL. **e**) Expression of autophagy-related genes analyzed by RT-qPCR in RAW264.7 cells in the different groups (*n* ≥ 3). (**f**) Western blot images of P62, LC3I/II, and GAPDH in RAW264.7 cells from different treatment groups. The experimental procedure was as follows: RAW264.7 cells were pretreated with CQ (5 µM) or 3-MA (5 mM) as required by experimental conditions for 2 h, followed by stimulation with fresh medium containing LPS (100 ng/mL) for 24 h. (**g**) Quantitative analysis of P62 and LC3-II/LC3-I ratio in RAW264.7 cells based on Western blot images (*n* ≥ 3). (**h**) Representative images from autophagic flux visualization assays using the mRFP-GFP-LC3 tandem fluorescence reporter system. Stable RAW264.7 cells expressing this reporter were seeded onto material surfaces. Depending on experimental conditions, cells were either pretreated with CQ (5 µM) or 3-MA (5 mM) for 2 h, followed by stimulation with fresh medium containing LPS (100 ng/mL) for 24 h. Autophagosomes (APs) appear as yellow puncta (mRFP^+^GFP^+^), whereas autophagolysosomes (ALs) exhibit only red fluorescent puncta (mRFP^+^GFP^–^). Scale bar = 20 μm. (**i**) Quantitative statistical analysis of the number of APs, number of ALs, and the AL/AP ratio derived from fluorescence images (*n* ≥ 3). *P < 0.05, **P < 0.01, ***P < 0.001, and ****P < 0.0001

Advancements in platforms such as liquid chromatography-tandem mass spectrometry (LC-MS/MS) have enhanced the sensitivity, accuracy, and throughput of proteomics research. These technological advancements provide critical insights into cellular functions, signal transduction pathways, and disease mechanisms [34]. Herein, we employed Data-Independent Acquisition (DIA)-based quantitative proteomics to identify the molecular pathways regulated by AE-MXene (Fig. 4a). Principal component analysis (PCA) confirmed distinct protein expression profiles among groups (Figure S6). In this study, we specifically examined changes in protein expression in macrophages treated with AE-MXene compared to those treated with TA under LPS-induced inflammatory stimulation. Proteins exhibiting a fold change greater than two and a p-value less than 0.05 were defined as significantly differentially expressed. Volcano plot analysis revealed that AE-MXene treatment led to the upregulation of 39 proteins and downregulation of 48 proteins (Fig. 4b). Functional enrichment analysis highlighted autophagy as a key pathway (Fig. 4c)—a finding consistent with the well-established critical role of autophagy in maintaining macrophage homeostasis and mediating stress responses [35, 36]. LC3 is a well-recognized hallmark of autophagy activation, while degradation of p62/sequestosome 1 (SQSTM1) denotes autophagic flux and protein degradation [37]. Heatmap and RT-qPCR analyses verified upregulation of autophagy-related genes (Ulk1, Beclin1, Atg5, Map1lc3) and downregulation of p62 (Sqstm1) (Fig. 4d and e). Collectively, these findings suggest that AE-MXene modulates the immune microenvironment by regulating the expression of autophagy-related proteins in macrophages.

To further elucidate the regulatory role of AE-MXene in macrophage autophagy, we utilized two pharmacological inhibitors targeting distinct stages of the autophagic pathway: chloroquine (CQ), which inhibits lysosomal degradation, and 3-methyladenine (3-MA), which blocks autophagosome biogenesis. Western blot analysis confirmed that AE-MXene treatment enhanced the LC3-II/LC3-I ratio and promoted p62 degradation, which is indicative of autophagic induction. CQ treatment significantly elevated both the LC3-II/LC3-I ratio and p62 accumulation, confirming its role in blocking late-stage autophagic flux. Conversely, 3-MA treatment markedly reduced the LC3-II/LC3-I ratio, consistent with its inhibition of the early autophagic stage (Fig. 4f and g). Live-cell imaging using mRFP-GFP-LC3 lentivirus further validated the activation of complete autophagic flux by AE-MXene: it increased the number of yellow (autophagosome) and red (autolysosome) puncta, whereas CQ blocked flux (resulting in accumulated yellow puncta) and 3-MA inhibited puncta formation (Fig. 4h and i). Notably, the use of broad-spectrum inhibitors (CQ, 3-MA) may introduce potential off-target effects (e.g., interference with endocytosis or apoptosis), which should be acknowledged as a limitation of this study. Future studies will utilize CRISPR/Cas9-mediated knockout of Atg5 or Beclin1 to validate the autophagy-specific regulatory mechanisms.

In summary, these complementary experimental approaches demonstrate that AE-MXene effectively induces and enhances complete autophagic flux in macrophages. Furthermore, the inhibitory effects of both CQ and 3-MA confirm that AE-MXene-induced autophagy can be effectively abrogated at multiple stages of the autophagic pathway.

### AE-MXene enhances osteogenic and angiogenic differentiation via macrophage autophagy

**Fig. 5:**
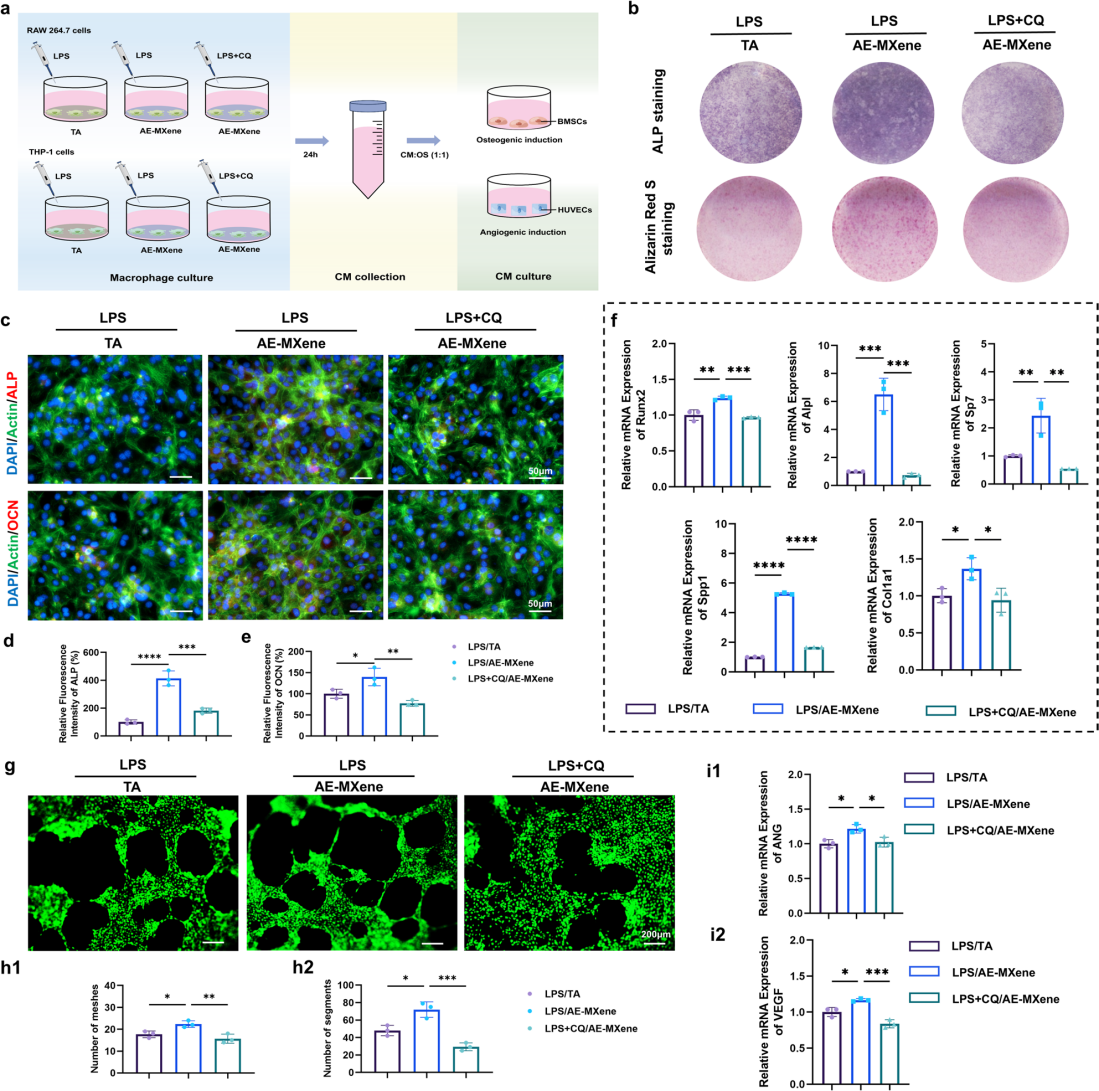
AE-MXene enhanced osteogenic and angiogenic differentiation by inducing macrophage autophagy. **a**) Schematic diagram of the conditional culture. RAW264.7 and THP-1 cells were pretreated with CQ (5µM) for 2 h and then stimulated with fresh medium containing LPS for 24 h, and the supernatant was collected. **b**) ALP staining images, Alizarin Red S staining images; **c**) merged immunofluorescence staining images of different experimental groups under conditional culture conditions. Scale bar = 50 μm. (**d**, **e**) Quantitative analysis of ALP and OCN in BMSCs in the different groups with the conditional culture (*n* ≥ 3). **f**) Expression of osteogenesis-related genes analyzed by RT-qPCR in BMSCs in the different groups with the conditional culture (*n* ≥ 3). **g**) Immunofluorescent staining images of HUVECs tubule formation in the different groups with the conditional culture. Scale bar = 200 μm. (**h**1–2) Quantitative analysis of the number of meshes and segments in immunofluorescence images (*n* ≥ 3). (**i**1–2) Expression of angiogenesis-related genes analyzed by RT-qPCR in HUVECs in the different groups with the conditional culture (*n* ≥ 3). *P < 0.05, **P < 0.01, ***P < 0.001, and ****P < 0.0001

Macrophage autophagy is well-documented to promote M2 polarization, mitigate inflammation, and support tissue regeneration [38, 39]. To validate the role of autophagy in AE-MXene’ s biological effects, we used macrophage-conditioned medium (CM) to simulate paracrine interactions between macrophages and target cells (Fig. 5a).

ALP staining and Alizarin Red S staining demonstrated that AE-MXene significantly enhanced the osteogenic potential of BMSCs compared with TA. Notably, pretreatment of cells with the autophagy inhibitor chloroquine (CQ) reversed this osteogenic-promoting effect (Fig. 5b). Immunofluorescence analysis further confirmed that AE-MXene upregulated the expression of early (ALP) and mid-stage (osteocalcin, OCN) osteogenic markers in BMSCs. CQ pretreatment significantly attenuated the AE-MXene-induced upregulation of these markers (Fig. 5c and d, and 5e). RT-qPCR analysis further verified that CQ treatment reduced the expression of key osteogenic genes (Runx2, Alpl, Sp7, Spp1, Col1a1) in BMSCs (Fig. 5f). These findings indicate that AE-MXene enhances BMSC osteogenic differentiation under inflammatory conditions by promoting macrophage autophagy.

Consistent with the osteogenic findings, tubule formation assays (a surrogate for angiogenic capacity) demonstrated that AE-MXene’ s pro-angiogenic effects were abrogated by CQ pretreatment (Fig. 5g and h). Additionally, RT-qPCR analysis revealed that AE-MXene upregulated the expression of angiogenesis-related genes (e.g., ANG, VEGF) in endothelial cells, whereas CQ pretreatment suppressed this AE-MXene-induced upregulation (Fig. 5i). Collectively, our findings innovatively demonstrate that, under inflammatory conditions, AE-MXene promotes angiogenic differentiation by regulating macrophage autophagy. While these results support autophagy as a key mediator of AE-MXene’ s biological effects, the non-specificity of CQ and 3-MA remains a limitation; future studies will employ autophagy-related gene silencing (e.g., Atg5 small interfering RNA (siRNA)) to eliminate off-target effects.

### AE-MXene enhances macrophage autophagy via the AMPK-mTOR pathway

**Fig. 6:**
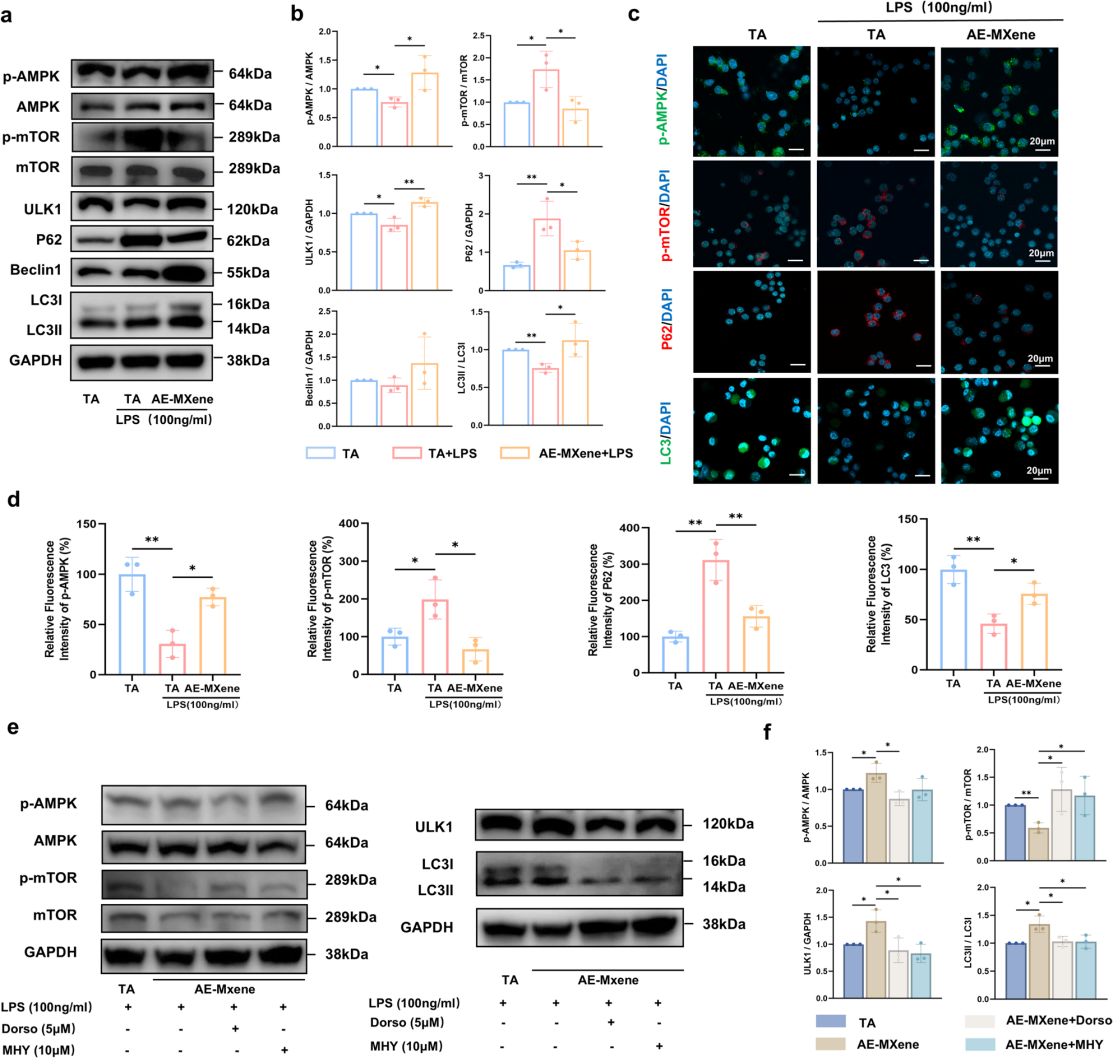
AE-MXene enhanced macrophage autophagy via activation of the AMPK-mTOR signaling pathway. **a**) Western blot images of p-AMPK, AMPK, p-mTOR, mTOR, ULK1, P62, Beclin1, LC3I/II, and GAPDH in RAW264.7 cells in different treatment groups. **b**) Quantitative analysis of p-AMPK/AMPK ratio, p-mTOR/mTOR ratio, ULK1, P62, Beclin1, and LC3-II/LC3-I ratio in RAW264.7 cells based on Western blot images (*n* ≥ 3). **c**) Immunofluorescence staining images of p-AMPK, p-mTOR, P62, and LC3 in RAW264.7 cells in different treatment groups. Scale bar = 20 μm. **d**) Immunofluorescence staining analysis of different treatment groups (*n* ≥ 3). (e) RAW264.7 cells were pretreated with dorsomorphin (5µM) and MHY1485 (10µM) as required and then stimulated with fresh medium containing LPS for 24 h. Western blot images of p-AMPK, AMPK, p-mTOR, mTOR, ULK1, LC3I/II, and GAPDH in RAW264.7 cells in different treatment groups. (f) Quantitative analysis of p-AMPK/AMPK ratio, p-mTOR/mTOR ratio, ULK1, and LC3-II/LC3-I ratio in RAW264.7 cells based on Western blot images (*n* ≥ 3). *P < 0.05, **P < 0.01

Adenosine monophosphate-activated protein kinase (AMPK) is a central energy sensor that regulates macrophage polarization and autophagy by inhibiting the mechanistic target of rapamycin (mTOR) [40–43]. Western blot analysis revealed that, under LPS stimulation, AE-MXene reversed LPS-induced perturbations: it increased the p-AMPK/AMPK ratio, decreased the p-mTOR/mTOR ratio, upregulated ULK1 expression and the LC3-II/LC3-I ratio, and downregulated p62 (Fig. 6a and b). Immunofluorescence staining further confirmed elevated p-AMPK and LC3 levels, as well as reduced p-mTOR and p62 expression, in the AE-MXene group (Fig. 6c and d).

**Fig. 7:**
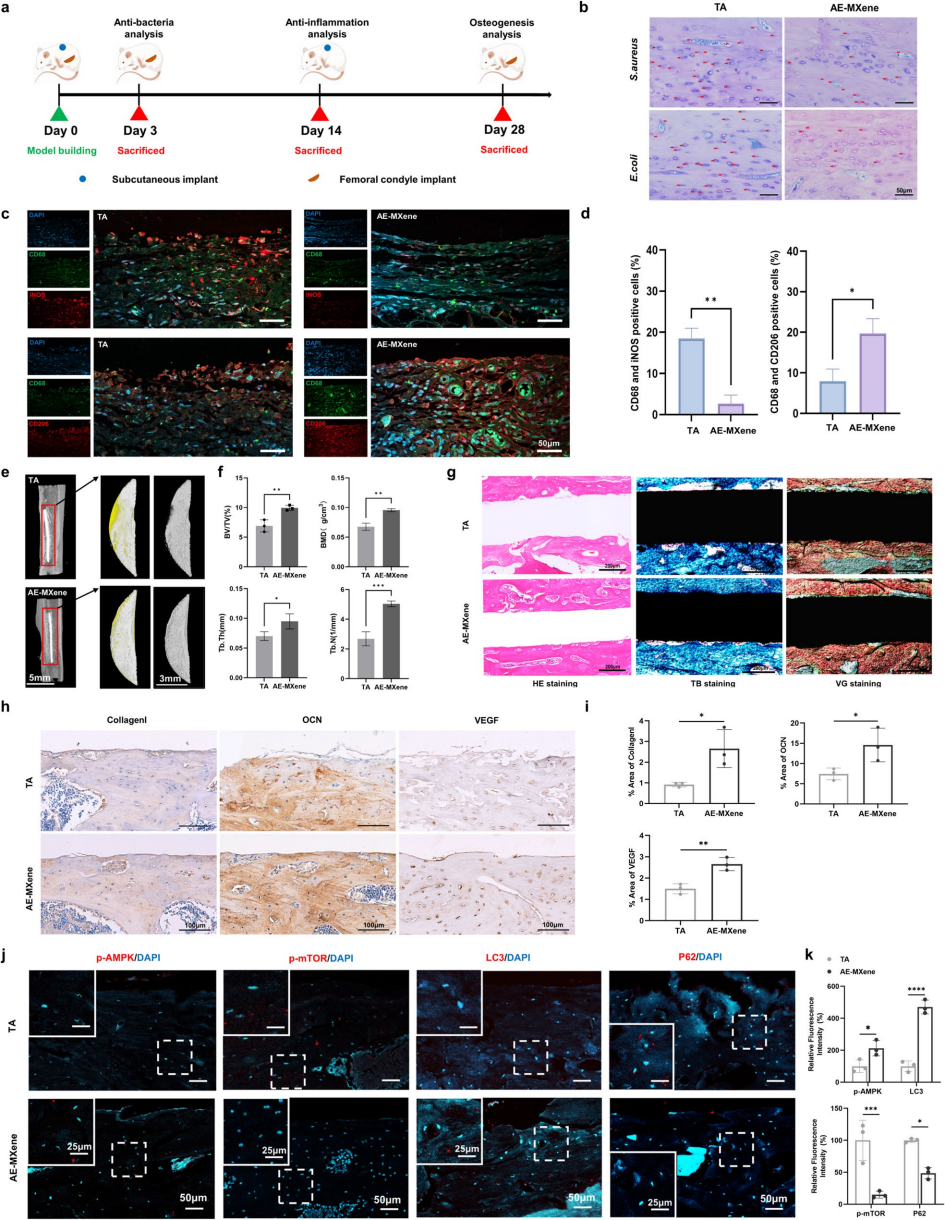
In vivo therapeutic efficacy and mechanism of AE-MXene. Panels are arranged chronologically according to the experimental sequence: antibacterial model (**b**), anti-inflammatory model (**c**, **d**), osseointegration model (**e**–**i**), and in vivo mechanistic signaling (**j**, **k**). **a**) Schematic diagram of the in vivo evaluation. **b**) Representative images of Giemsa staining for in vivo antibacterial evaluation. Scale bar = 50 μm. (**c**, **d**) Representative images and quantitative analysis of immunofluorescent staining for macrophages in subcutaneous tissue. *n* ≥ 3. Scale bar = 50 μm. **e**) Three-dimensional micro-CT reconstructions of the defect site. New bone is shown in grey, and the titanium implant is pseudo-colored in yellow for clarity. Scale bar = 5 μm (first column); 3 μm (second and third column). **f**) The micro-CT analysis showed bone volume fraction (BV/TV), bone mineral density (BMD), trabecular thickness (Tb.Th), and trabecular number (Tb.N). *n* ≥ 3. g) HE, TB, and Van Gieson (VG) staining images of femoral condyle defect slides. Scale bar = 200 μm. (**h**, **i**) Collagen I, OCN, and VEGF immunohistochemical staining images and quantitative analysis for TA and AE-MXene samples after removal of titanium rods. Specific staining was confirmed using negative controls with non-immune IgG, shown in Supplementary Figure S15. *n* ≥ 3. Scale bar = 100 μm. (**j**, **k**) Representative images and quantitative analysis of immunofluorescent staining of p-AMPK, AMPK, LC3, and P62 in femoral condyle defect. *n* ≥ 3. Scale bar = 50 μm (large box); 25 μm (small box). *P < 0.05, **P < 0.01, ***P < 0.001, and ****P < 0.0001

To further confirm the involvement of the AMPK-mTOR pathway in AE-MXene-mediated autophagy, we conducted validation experiments using Dorsomorphin (an AMPK inhibitor) and MHY1485 (an mTOR activator). Western blot results showed that following AE-MXene-induced autophagy activation, Dorsomorphin treatment significantly reduced the p-AMPK/AMPK ratio, increased the p-mTOR/mTOR ratio, and concurrently decreased ULK1 expression and the LC3-II/LC3-I ratio. Similarly, MHY1485 treatment enhanced the p-mTOR/mTOR ratio and reduced ULK1 expression and the LC3-II/LC3-I ratio, thereby counteracting the autophagy-promoting effect of AE-MXene (Fig. 6e and f). Furthermore, immunofluorescence analysis of bone tissue sections from rat femoral implant models corroborated these in vitro findings: the fluorescence intensities of p-AMPK and LC3 in the AE-MXene group were significantly higher than those in the TA group, while the expression levels of p-mTOR and p62 were significantly lower (Fig. 7j and k). To the best of our knowledge, this study is the first to demonstrate that the two-dimensional AE-MXene enhances macrophage autophagy via the AMPK-mTOR pathway, thereby providing a molecular basis for the rational design of autophagy-targeted implants.

### AE-MXene exhibits anti-inflammatory, osteogenic, and angiogenic effects *in vivo*

The in vivo therapeutic efficacy of AE-MXene was assessed using subcutaneous and femoral implantation models in rats, as illustrated in Fig. 7a. All experimental animals were healthy at the designated endpoint, with no mortality observed. Histological examination of major organs revealed no overt pathological lesions (Figure S7). Concurrently, biochemical assays of hepatic and renal function markers in cardiac blood yielded values within the normal physiological range (Figure S8), confirming the biocompatibility of AE-MXene.

To assess the anti-inflammatory efficacy of AE-MXene at the implant site, we evaluated macrophage polarization via immunofluorescence staining for pro-inflammatory M1 macrophage marker iNOS and anti-inflammatory M2 macrophage marker CD206. In the TA group, widespread iNOS expression was accompanied by the formation of irregular fibrous layers, whereas CD206 expression was relatively low. In contrast, the AE-MXene group exhibited a marked reduction in CD68⁺/iNOS⁺ (M1) macrophages and a significant increase in CD68⁺/CD206⁺ (M2) macrophages (Fig. 7c and d). These findings demonstrate that AE-MXene effectively mitigates peri-implant inflammation and fosters an anti-inflammatory microenvironment.

To evaluate the in vivo osteogenic effect of AE-MXene, implants were inserted into the distal femur of rats and maintained for 4 weeks. The intraoperative schematic of the femoral implantation model (Figure S9) indicates successful implant placement, highlighted by the white arrows. Three-dimensional (3D) reconstruction of the implant and surrounding bone tissue (Fig. 7e) revealed increased new bone formation in the AE-MXene group. Quantitative analysis demonstrated that bone volume fraction (BV/TV), bone mineral density (BMD), trabecular thickness (Tb.Th), and trabecular number (Tb.N) were all significantly higher in the AE-MXene group (Fig. 7f).

Histological analysis provided further evidence of superior osseointegration. Hematoxylin and eosin (H&E) staining showed direct bone apposition to the AE-MXene implant surface (without an intervening fibrous tissue layer), osteoblasts embedded within the adjacent new bone matrix, and reduced inflammatory cell infiltration at the bone-implant interface; Toluidine blue (TB) staining revealed a continuous band of metachromatic (blue) staining, indicative of mature, mineralized bone in direct contact with the implant; Van Gieson (VG) staining demonstrated more extensive and continuous deposition of red-stained mature collagenous bone (rich in collagen type I) at the AE-MXene interface. These histomorphometric observations confirm more direct structural contact and the formation of a mature, mineralized bone matrix in the AE-MXene group (Fig. 7g). Furthermore, immunohistochemical analysis of bone tissue sections revealed a significant upregulation in the expression of key osteogenic (Collagen I, OCN) and angiogenic (VEGF) markers in the AE-MXene group (Fig. 7h and i). These in vivo results demonstrate that AE-MXene possesses robust osteogenic and angiogenic properties, corroborating the positive effects observed in prior in vitro studies.

It should be noted that this study aimed to investigate the initial biological responses to the AE-MXene coating. The 4-week observation period was suitable for evaluating early cellular activities and the initial stages of bone formation but does not encompass the later phases of bone remodeling. Additionally, while significant improvements in bone volume fraction and bone-implant contact provide strong morphological evidence for implant stability, these are correlative measures rather than direct assessments of functional mechanical integration. Therefore, subsequent studies should adopt a longer observation period (8–12 weeks) and incorporate functional biomechanical analyses to validate the long-term stability and functional strength of the AE-MXene-coated bone-implant interface.

### AE-MXene exhibits antibacterial effects *in vitro* and *in vivo*

AE-MXene exhibited potent antibacterial activity against Staphylococcus aureus (*S. aureus*) and Escherichia coli (*E. coli*): live/dead bacterial staining and plate-counting assays demonstrated significantly reduced bacterial viability (74.5% and 52.5% for *S. aureus* and *E. coli*, respectively) and colony-forming unit (CFU) counts (57.8% and 57.3% reduction) compared to the TA group (Figure S10 and S11).

To elucidate the underlying antibacterial mechanism of AE-MXene, we conducted supplementary experiments focusing on oxidative stress induction and cell membrane integrity. The intracellular reactive oxygen species (ROS) levels in *S. aureus* and *E. coli* were quantified via the fluorescent probe 2’,7’-dichlorodihydrofluorescein diacetate (DCFH-DA). The results revealed a significant increase in ROS accumulation in both bacterial strains following AE-MXene treatment, that AE-MXene induces potent oxidative stress in bacteria (Figure S12). Concurrently, we evaluated bacterial cell membrane damage: a bicinchoninic acid (BCA) assay was used to quantify bacterial protein leakage, and ultraviolet absorption at 260 nm was employed to measure nucleic acid release. Both assays demonstrated significantly elevated levels of these cytoplasmic components in the supernatants of AE-MXene-treated *S. aureus* and *E. coli* cultures (Figure S13). This direct evidence of protein and nucleic acid leakage substantiates that AE-MXene disrupts bacterial cell membrane integrity.

The in vivo antibacterial performance of AE-MXene was further validated using a rat femoral implantation model. Implants pre-coated with pre-formed *S. aureus* and *E. coli* biofilms were implanted into the distal femurs of rats. Three days post-implantation, H&E and Giemsa staining were performed to visualize residual bacteria and tissue inflammatory responses. Compared to the TA group, the AE-MXene group exhibited significantly reduced inflammatory cell infiltration and more intact bone tissue architecture (Figure S14). Additionally, the AE-MXene group contained significantly fewer residual bacteria (indicated by red arrows) than the TA group (Fig. 7b). Collectively, these results confirm that AE-MXene exhibits superior antibacterial activity in both in vitro and in vivo settings.

## Conclusion

In this study, we developed a novel surface modification strategy by integrating AE technology with MXene nanosheet coatings technology to fabricate AE-MXene titanium implants. This approach facilitates the uniform and stable anchoring of MXene onto titanium substrates, resulting in the formation of a micro/nanostructured surface with substantially enhanced roughness and hydrophilicity. In vitro and in vivo investigations verified that AE-MXene possesses multiple bioactive functions, including osseointegration promotion, anti-inflammatory activity, and antibacterial efficacy. Notably, this study is the first to demonstrate that AE-MXene induces macrophage autophagy via the AMPK-mTOR pathway, which in turn facilitates osteogenesis and angiogenesis. In conclusion, this work describes a simple yet effective surface modification strategy for orthopedic titanium implants, while offering valuable mechanistic insights into how implant coatings can modulate osteogenesis and angiogenesis to improve clinical outcomes.

## Experimental section

### Materials

Ti6Al4V sheets were purchased from Hefei Wenghe Metal Materials Co., Ltd (China). Minimum essential medium (MEM-), dulbecco’s modified eagle medium (DMEM), and roswell park memorial institute (RPMI) were purchased from Gibco BRL (USA). Angiogenic induction medium, matrigel, and fetal bovine serum (FBS) were purchased from OriCell (Guangzhou, China). Penicillin and streptomycin were purchased from Thermo Scientific (Waltham, MA, USA). CCK-8 was purchased from Proteintech (Wuhan, China). Calcein/PI cell viability/cytotoxicity kit, ALP assay kit, BCIP/NBT ALP color development kit and Alizarin Red S Staining Kit for Osteogenesis were bought from Beyotime Biotechnology (Shanghai, China). Bacterial lipopolysaccharide (LPS, Escherichia coli) was obtained from PeproTech (USA). Dorsomorphin dihydrochloride (Dorso) was obtained from TargetMol Chemicals Inc. (USA). Chloroquine (CQ), 3-Methyladenine (3-MA), and MHY1485 (MHY) were purchased from MCE (Shanghai, China). FITC-conjugated phalloidin was purchased from Merck (USA). The Prime Script RT Reagent Kit and SYBR Premix Ex Taq II were purchased from Takara Biotechnology (Shiga, Japan). Primary antibodies against GAPDH, DAPI, ALP, OCN, and secondary antibodies were purchased from Cell Signaling Technology (Danvers, MA, USA). Primary antibodies against AMPK, LC3B, CD68, iNOS, CD206, and VEGF were purchased from Proteintech (Wuhan, China). Primary antibodies against phosphor-AMPK, mTOR, phosphor-mTOR, ULK1, p62/SQSTM1, Beclin1, ATG5, and Collagen I were obtained from AbMart Bio-tech (Shanghai, China).

### Fabrication and characterization of MXene

To prepare MXene, 2 g of Ti₃AlC₂ powder was added to a mixture of 3.2 g LiF in deionized water and 12 M HCl, followed by etching at 40 °C for 48 h. The solution was centrifuged and washed until the pH reached 6–7, continuing centrifugation until the solution turned dark green or black and became uniform. The solution was then treated with nitrogen and ultrasonic agitation at 40 kHz, 200 W for 1 h in a water bath. The resulting MXene dispersion was protected by nitrogen and stored at low temperatures. The MXene dispersion was stored at low temperatures. MXene dispersion was diluted to concentrations of 0.025, 0.05, 0.1, and 0.2 mg/mL, and ultrasonic treatment was applied for 15 min. Zeta potential tests were performed using a solid-surface zeta potential tester (SurPASS3 Eco, Anton Paar, Austria). Dynamic light scattering (DLS) particle size detection tests were performed using a DLS tester (Malvern Zetasizer Nano ZS, Malvern Instruments, UK).

### Alkali etching of titanium sheet

Titanium (Ti6Al4V) sheets: 0.2 mm thickness for in vitro studies and 0.1 mm thickness for in vivo studies. The sheets were polished with metallographic sandpaper, cleaned in acetone, ethanol, and deionized water, and dried with a nitrogen stream. The pre-cleaned titanium sheets were immersed in the KOH solution (4 M) for 1, 2, 4, or 8 h, to investigate the effect of etching time on surface morphology. The etching was performed in a thermostatically controlled water bath (Model: HWS-35, VWR) with continuous stirring, then washed in deionized water, cleaned ultrasonically, and dried.

### Cell cultures

Bone marrow mesenchymal stem cells (BMSCs) were obtained and cultured as previously described [44]. This study was approved by the Animal Care and Experiment Committee of the Ninth People’s Hospital (SH9H-2024-A1434-1). Primary BMSCs were suspended in MEM-α with 10% FBS and 1% penicillin/streptomycin, and third-generation BMSCs were used for experiments. HUVECs were obtained from OriCell (Shanghai, China) and cultured in the angiogenic induction medium and matrigel. The RAW 264.7 cell line and THP-1 cell line were derived from Stem Cell Bank, Chinese Academy of Sciences (Shanghai, China). RAW 264.7 cells were cultured in DMEM (adding 5% FBS and 1% penicillin/streptomycin), while THP-1 cells were cultured in RPMI 1640 (adding 10% FBS and 1% penicillin/streptomycin).

*4.5. CCK-8 Assay*: The CCK-8 kit was used to determine cytotoxicity. BMSCs, HUVECs, and RAW264.7 cells were inoculated onto different types of titanium sheets (repeat three times). At the specified number of days, add CCK-8 reagent at a ratio of 1:10 to each well and continue to culture for 4 h. The absorbance value was measured at 450 nm.

### Live/Dead cell staining

The Calcein/PI cell viability kit was used to evaluate cell viability. BMSCs, HUVECs, and RAW264.7 cells were inoculated onto different types of titanium sheets and repeated thrice. After culture for the designated time points, the culture medium was removed, and the cells were washed thoroughly with PBS. A detection working solution (Calcein-AM 1:1000, PI 1:1000) was added, and the samples were incubated at 37 °C for 30 min. The stained cells were observed using a fluorescence microscope (Leica, Germany). Viable cells stained with Calcein-AM fluoresced green, while dead cells with compromised membranes stained with PI fluoresced red.

### Fabrication and surface characterization of AE-MXene

AE-MXene specimens were obtained by applying 0.1 mg/mL MXene solution on the alkali-etched titanium sheet and drying at 50℃ for 4 h. Morphological analysis was conducted using FE-SEM (Hitachi S-4800, Japan). To evaluate the crystal structures, XRD patterns were recorded with a D/Max-2550 PC (Rigaku, Japan). The chemical composition and states were analyzed through XPS (ESCALAB 250, Thermo Scientific, USA). Additionally, AFM (Keyence, Japan) was used to examine the surfaces of AE-MXene specimens. The wettability of the scaffolds was assessed using a contact angle goniometer (TL101, Biolin Scientific AB). A droplet of ultrapure water was deposited on the material surface in static mode for the contact angle measurement. The wettability was then evaluated by observing the angle formed between the water droplet and the material.

### Flowcytometry for macrophage

RAW 264.7 cells were plated onto 6-well plates containing sterilized materials (3 replicates per group) and cultured until 80% confluency. The cells were then stimulated with LPS (100 ng/mL) for 24 h. After the culture was completed, the cells were collected, and antibodies against F4/80, anti-CD11B, anti-CD86 (M1), and anti-CD206 (M2) were added. The expression levels of the markers were detected by FACScan flow cytometer (BD Biosciences, CA). The data were processed using FlowJo software to calculate the polarization of the cells.

### Quantitative real-time PCR (qRT-PCR) analysis

 Cultivate cells according to different requirements. To detect the level of inflammatory suppression, macrophages were cultured under LPS-induced inflammatory stimulation for 24 h. BMSCs were cultured under osteogenic induction conditions to detect the osteogenic level for 3, 7, and 14 days. HUVECs were cultured under angiogenic cell induction conditions to detect the angiogenic level. The total RNA of each group was extracted using TRIzol (Invitrogen, USA), and cDNA transcription was performed on cells using The Prime Script RT Reagent Kit. qRT-PCR was carried out using the Light Cycler­480 Instrument II (Roche, Basel, Switzerland) with SYBR Premix Ex Taq II. Reaction system: 5µL SYBR green master mix, 3 µL double-distilled water (ddH2O), 1µL primer and 1µL cDNA. GAPDH is an internal reference gene, and relative gene expression levels were determined using the 2⁻ΔΔCt method. Each group has three repetitions. Primer sequence information can be found in (Supplementary material: Table 1).

### ALP activity detection

BMSCs were inoculated onto different titanium sheets and cultured in normal and bone-induced media (Oricell, China). On the seventh day, the cells were lysed with RIPA lysis buffer (Beyotime, China) (without inhibitors), the cell supernatant was collected, and the ALP activity was determined. According to the manufacturer’s instructions for the ALP assay kit, samples were added to the chromogenic substrate solution and incubated at 37 °C for 30 min. The absorbance was measured at 405 nm. The total Protein content of each group was detected by the BCA Protein Assay Kit (Beyotime, China), and the ALP level was normalized. Three experiments were conducted for each group of samples.

### Angiogenesis experiment

For different detection purposes, matrigel coating was placed in 24-well plates with or without sterilized materials (3 replicates per group), with 250 µL per well, and gelated at 37 °C for 30 min. HUVECs were starved for 8 h. HUVECs were seeded onto matrigel at a density of 1.5 × 10^5^ cells per well. Six hours later, Calcein-AM was introduced to the cells at a final concentration of 2ug/ml and incubated at 37 degrees in the dark for 30 min. The tubular structure images were captured using an inverted fluorescence microscope (Leica, Germany), and the parameters, such as the number of branch nodes and the length of the main pipe formed, were analyzed using ImageJ software (National Institutes of Health).

### Mass spectrometry and bioinformatics analysis

To explore the regulatory effects of different materials on the protein expression profiles of macrophages, RAW 264.7 cells were inoculated into different groups of materials (3 replicates), and the cells were collected after 24 h of LPS stimulation. Total protein was extracted using the lysis buffer. The concentration was measured using the BCA method and uniformly standardized before being sent to the mass spectrometry platform Bioprofile (Shanghai, China) for liquid chromatography-tandem mass spectrometry (LC-MS) analysis. Take an appropriate amount of protein from each sample for FASP enzymatic hydrolysis, respectively, and the peptide segments are enriched and desalinated by a C18 column. All mass spectrometry data were merged through the software Spectronaut to complete the database retrieval of DIA mass spectrometry data and the quantitative analysis of protein DIA. Screen the differentially expressed proteins (Fold change ≥ 2, *p* < 0.05), and perform GO functional annotation and KEGG pathway enrichment analysis to reveal the changes in the related signaling pathways induced by the materials.

### Western blot assay

For different detection purposes, once RAW264.7 cells cultured on each group of materials reached 80% confluence, they were pretreated with specific pretreatment agents: CQ (5 µM, late-stage autophagy inhibitor), 3-MA (5 mM, early-stage autophagy inhibitor), Dorso (5 µM, phosphorylated AMPK inhibitor), and MHY (10 µM, phosphorylated mTOR activator). Replace the fresh medium containing LPS (100 ng/mL) and induce for 24 h.

Total protein of the cells was extracted, and the protein concentration was measured using the BCA assay kit (Beyotime Biotechnology, China). Equal amounts of protein were separated on SDS-polyacrylamide gels (GenScript, China). Subsequently, the protein was separated by electrophoresis, transferred to a PVDF membrane, and then blocked for 1 h. Specific primary antibodies were added respectively and incubated overnight. Add the corresponding secondary antibody the next day and incubate for 1 h. Imaging was performed using the Odyssey M Imaging System (Li-Cor Biosciences, USA). ImageJ analyzes the gray values, and GAPDH normalizes for the internal parameters.

### Assessment of autophagic flux using an mRFP-GFP-LC3 reporter system

To dynamically monitor autophagic flux, a stable RAW 264.7 macrophage cell line expressing the autophagy reporter mRFP-GFP-LC3 was generated. At 50–70% confluence, cells were transduced with the mRFP-GFP-LC3 lentivirus (Han Heng Biotechnology, China) at a multiplicity of infection (MOI) of 30. Polybrene (5 µg/mL) was added to enhance transduction efficiency. After 6 h, the viral medium was replaced with fresh complete medium. Transfection efficiency was confirmed by fluorescence microscopy 48–72 h post-transduction, based on the co-expression of GFP and mRFP. To select for stably transduced cells, culture medium containing puromycin (5 µg/mL) was applied. Selection continued until all untransfected control cells died, leaving a stably proliferating population. This stable cell line was expanded and cryopreserved for subsequent experiments.

For autophagic flux experiments, the stable reporter cells were seeded onto different material surfaces (*n* = 3 wells per group). Upon reaching 80% confluence, cells were pre-treated for 2 h with the autophagy inhibitors CQ (5 µM) or 3-MA (5 mM) as required by the experimental design. Following pre-treatment, cells were stimulated for 24 h with fresh medium containing LPS (100 ng/mL). After treatment, cells were gently washed twice with ice-cold PBS. Images were acquired directly using CLSM. Quantitative analysis was performed using ImageJ software. Autophagosomes were identified as yellow puncta (mRFP^+^GFP^+^), while autolysosomes were identified as red puncta (mRFP^+^GFP^−^). Autophagic flux activity was quantified by calculating either the ratio of red to yellow puncta or the corresponding fluorescence intensities.

### Collection of conditioned mediums (CM)

RAW264.7 cells and THP-1 cells differentiated by PMA were inoculated onto different material groups. After the cells adhered, chloroquine (CQ, 5µM) was added for pretreatment for 4 h, and then fresh medium containing LPS (100 ng/mL) was replaced and stimulated for 24 h. After the experiment, the supernatants of each group were collected, and the centrifuge was used for 10 min to remove cell debris and obtain the conditioned medium. The culture medium was immediately aliquoted and stored at −80 °C for subsequent experiments.

### ALP staining and Alizarin red S (ARS) staining

BMSCs were inoculated in 24-well plates. After the cells adhered, they were replaced with each group of CM and continuously cultured for 7 days and 14 days. The fresh CM should be replaced during culture every 2 to 3 days. On day 7, cells were washed with PBS, fixed with 4% paraformaldehyde for 15 min at room temperature, and stained using a BCIP/NBT ALP color development kit according to the manufacturer’s instructions. The staining reaction was incubated for 30 min at room temperature before being terminated and washed with PBS (*n* = 3). On day 14, cells were washed, fixed as described above, and stained using an Alizarin Red S staining kit for osteogenesis per the manufacturer’s protocol. The ARS solution was incubated for 30 min at room temperature. The reaction was then terminated, and the wells were washed repeatedly with PBS (*n* = 3).

### Immunofluorescence for osteoblasts

BMSCs was inoculated into 24-well plates (3 replicates), adhered to the plates, replaced with each group of CM, and cultured for 7 days. Fresh CM should be replaced every 2 to 3 days during the culture period. On the seventh day, after washing with PBS, it was fixed with 4% paraformaldehyde for 15 min, permeated with 0.1% Triton X-100 for 10 min, and then blocked for 1 h. Add the primary antibodies Actin, ALP, and OCN and incubate overnight. The next day, add the corresponding fluorescent-labeled secondary antibody (Alexa Fluor 488 or 594) and incubate in the dark for 1 h. After re-staining the cell nuclei with DAPI, images were collected using confocal laser scanning microscopy (CLSM; LSM880, ZEISS, Germany). Fluorescence intensity was quantified using ImageJ software.

### Immunofluorescence for macrophages

To assess the effects of different materials on macrophage autophagy and related signaling pathways, RAW 264.7 cells were seeded onto the material surfaces (*n* = 3 replicates per group). Upon reaching 80% confluency, the culture medium was replaced with fresh medium containing LPS (100 ng/mL) for 24 h to induce an inflammatory response. Following induction, the cells were fixed, permeabilized, and blocked. For immunofluorescence staining, cells were incubated with the following primary antibodies (all at 1:200 dilution): anti-LC3, anti-p62, anti-p-AMPK, and anti-p-mTOR. After washing, the corresponding fluorescent secondary antibodies (Alexa Fluor 488 or 594, 1:500 dilution) were applied. Cell nuclei were counterstained with DAPI. Images were acquired using CLSM. Fluorescence intensity and protein localization were subsequently analyzed using ImageJ software.

### In vivo antibacterial evaluation

The Animal Care and Experiment Committee of the Ninth People’s Hospital, approved the animal protocol (SH9H-2024-A1434-1). All animal experiments complied with the American Psychological Association’s Guidelines for Ethical Conduct in the Care and Use of Nonhuman Animals in Research. 10-week-old male SD rats were used for in vivo studies. Prior to implantation, TA and AE-MXene samples (*n* = 5 per group) were incubated in bacterial suspensions of *S. aureus* and *E. coli* (1 × 10⁷ CFU/mL) for 24 h to induce bacterial biofilm formation. The surgical instrument used for defect creation was a 12 mm-diameter dental precision grinding circular saw with a thickness of 0.2 mm. During the operation, the positioning plane of the saw was maintained parallel to the long axis of the femur, and the thin edge of the rotating blade was used to create a precise sagittal-oriented wedge-shaped osteotomy on the femoral condyle. This osteotomy resulted in a thin slit-like defect with a maximum depth of 2 mm. The implants were angular segments cut from circular pure titanium sheets (12 mm in diameter, 0.2 mm in thickness), with a maximum width of 2 mm. This dimension was precisely tailored to match the size of the slit-like defect. During implantation, the planar implant was pressed into the defect to achieve a tight geometric fit, thereby avoiding poor fitting and artifact-induced inflammation. Three days after surgery, the rats were euthanized, and the relevant tissue samples were collected and fixed in 4% paraformaldehyde (PFA) for 48 h. Subsequent to decalcification and paraffin embedding, the samples were sectioned and stained with hematoxylin-eosin (H&E) and Giemsa to evaluate the infection status of peri-implant tissues.

### In vivo subcutaneous implant model

To assess the biocompatibility and inflammatory response of the new material, 10-week-old male SD rats were anesthetized. Longitudinal incisions were made on the back, and subcutaneous pockets were created for material implantation (*n* = 5 per group). The rats were euthanized two weeks later, and tissue samples were harvested for H&E staining and immunofluorescence analysis. CD68 identified macrophages, while iNOS and CD206 were used to label pro-inflammatory M1 and anti-inflammatory M2 macrophages, respectively.

### *In vivo* bone-implantation osteointegration assays

 Following previously reported methods, a femoral bone defect model was established in SD rats [45]. After anesthesia, a femoral bone defect was created as described in Sect. "Immunofluorescence for macrophages", and the corresponding implant materials (*n* = 5 per group) were implanted and the wound sutured. Four weeks post-surgery, cardiac puncture was performed on anesthetized rats to collect approximately 3 mL of venous blood, which was transferred into heparinized anticoagulant tubes. After centrifugation, the separated plasma was used to measure biochemical indicators, including alanine aminotransferase (ALT), aspartate aminotransferase (AST), and blood urea nitrogen (BUN), for the assessment of liver and kidney functions. Then, the rats were sacrificed. After separating the distal femur, heart, liver, spleen, lung, and kidney, they were fixed with 4% paraformaldehyde and stored in 75% alcohol for subsequent experiments.

Samples were decalcified, paraffin-embedded, and sectioned. H&E staining is used to evaluate the infection of the in vivo biological toxicity of materials. Immunohistochemistry was used to detect osteogenic and angiogenic markers (Collagen I, OCN, VEGF). Negative control staining was performed on adjacent sections using non-immune rabbit polyclonal IgG (Cat No. 30000-0-AP, Proteintech) at the same concentration as the primary antibody. Sections were deparaffinized, antigen-retrieved, and incubated with primary antibodies overnight, followed by HRP-labeled secondary antibodies, DAB staining (Solarbio Science & Technology, China), and hematoxylin counterstaining. Images were captured under Vectra 3 Automated Quantitative Pathology Imaging System (Akoya Biosciences, USA).

For immunofluorescence analysis, tissue sections were permeabilized and blocked, followed by overnight incubation with primary antibodies (LC3, P62, p-mTOR, p-AMPK). The next day, fluorescent secondary antibodies were applied, followed by DAPI nuclear staining. Imaging was performed using CLSM to assess protein expression and localization.

### Micro-CT scanning

High-resolution micro-computed tomography (micro-CT) scanning was performed using a µCT-100 system (SCANCO Medical AG, Switzerland) with an isotropic voxel size of 10 μm. The scan parameters were set to an X-ray energy of 70 kV, a current of 200 µA, and an exposure time of 300 ms per projection. Three-dimensional reconstructions were generated from the acquired projections for subsequent structural evaluation. The region of interest (ROI) for all quantitative analyses was defined as a uniform volumetric shell encapsulating the implant. Axially, this ROI spanned the entire implant length, extended by 0.5 mm of bone at both the proximal and distal ends, which corresponded to approximately 251 contiguous image slices. Within each axial slice (cross-sectionally), the ROI included all bone tissue within a 0.5 mm radial distance (approximately a 42-pixel expansion) from the external implant surface. The final three-dimensional ROI was derived from the superimposition of these axial and cross-sectional criteria. The following bone morphometric parameters were quantified within this volumetric ROI: bone volume fraction (BV/TV), bone mineral density (BMD), trabecular thickness (Tb.Th), and trabecular number (Tb.N).

### Hard-tissue section staining

After fixation, tissues were dehydrated, cleared with xylene, and embedded in polymethyl methacrylate. The embedded samples were then sectioned into 150 μm-thick slices using a hard tissue microtome (Model 310, EXAKT, Germany) and subsequently polished to a final thickness of approximately 40 μm. Toluidine Blue and Basic Fuchsin (TB) staining and Van Gieson (VG) staining were performed to assess bone integration with the implanted materials. These histological evaluations enabled detailed analysis of new bone formation and the quality of the material–bone interface in vivo.

### Antibacterial evaluation *in vitro*

The antibacterial efficacy of the materials was evaluated against Staphylococcus aureus (*S. aureus*, ATCC 25923) and Escherichia coli (*E. coli*, ATCC 25922) using a series of complementary assays to assess viability, adhesion, oxidative stress, and membrane integrity.

Live/Dead Staining: Bacterial viability on material surfaces was assessed qualitatively using the LIVE/DEAD BacLight bacterial viability kit (Invitrogen, USA). Bacteria in the logarithmic growth phase were incubated on material samples (*n* = 3 per group) for 24 h at 37 °C. After incubation, non-adherent bacteria were removed by gentle washing with sterile PBS. Samples were stained with the working dye solution in the dark for 15 min and then visualized using CLSM.

Quantitative Adhesion (CFU Assay): Bacterial adhesion was quantified via a colony-forming unit (CFU) assay. Material samples (*n* = 3) were placed in a 6-well plate, inoculated with a bacterial suspension, and incubated at 37 °C for 2 h. Each sample was then rinsed thoroughly with sterile PBS, transferred to a centrifuge tube containing PBS, and subjected to ultrasonic agitation for 5 min to detach adherent bacteria. The resulting suspension was serially diluted, plated on solid agar, and incubated overnight. Viable adherent bacteria were enumerated by counting CFUs.

Intracellular ROS Detection: The generation of reactive oxygen species (ROS) was measured using a fluorescent probe (DCFH-DA, Beyotime). After incubating bacteria with material samples for 12 h, the bacterial suspensions were collected, incubated with 10 µmol/L DCFH-DA at 37 °C in the dark for 30 min, washed, and resuspended in PBS. Fluorescence intensity was measured with excitation/emission wavelengths of 488 nm and 525 nm, respectively.

Membrane Integrity Assay: Membrane damage was evaluated by quantifying the leakage of intracellular components. Following a 12-h co-culture of bacteria with material samples, the supernatants were collected. The concentration of leaked protein was determined using an enhanced BCA protein assay kit (Beyotime). The absorbance of leaked nucleic acids (DNA/RNA) in the supernatant was measured using a multimode microplate reader (BioTek, USA).

### Statistical analysis

Statistical analyses were performed using SPSS software (version 22.0; SPSS Inc., USA). Data are presented as mean ± standard deviation (SD). Differences between the experimental and control groups were assessed using Student’s t-test. For comparisons involving multiple groups, one-way analysis of variance (ANOVA) followed by the Scheffé post hoc test was used. Statistical significance was defined as: *P<0.05, **P<0.01, ***P < 0.001 and ****P < 0.0001.

## Supplementary Information

Supplementary Material 1

Supplementary Material 2

## Data Availability

The datasets used and/or analysed during the current study are available from the corresponding author on reasonable request.

## Abbreviations

AE: Alkali-etched surfaces
AE-MXene: Alkali-etched MXene
AFM: Atomic force microscopy
ALP: Alkaline phosphatase
AMPK: AMP-activated protein kinase
ANG: Angiopoietin
ARS: Alizarin red S
BCA: Bicinchoninic acid
BMD: Bone mineral density
BMSCs: Bone marrow mesenchymal stem cells
BV/TV: Bone volume fraction
CCK-8: Cell Counting Kit-8
CFU: Colony-forming units
CM: Conditioned medium
CQ: Chloroquine
DLS: Dynamic light scattering
DMEM: Dulbecco’s modified eagle medium
E. coli: Escherichia coli
H&E: Hematoxylin and eosin
HUVECs: Human umbilical vein endothelial cells
LC-MS/MS: Liquid chromatography-tandem mass spectrometry
3-MA: 3-methyladenine
MFI: Mean fluorescence intensity
MS: Mass spectrometry
mTOR: Mechanistic target of the rapamycin
MXene: Transition metal carbides and nitrides
OCN: Osteocalcin
PCA: Principal component analysis
qRT-PCR: Quantitative real-time PCR
S. aureus: Staphylococcus aureus
SEM: Scanning electron microscopy
TB staining: Toluidine blue and basic fuchsin staining
Tb.N: Trabecular number
Tb.Th: Trabecular thickness
VEGF: Vascular endothelial growth factor
VG staining: Van gieson staining
XPS: X-ray photoelectron spectroscopy
XRD: X-ray diffraction
