# Supplementary material for: AE-MXene-modified titanium alloy promotes osseointegration by regulating the AMPK-MTOR-autophagy pathway in macrophage
Source: J Nanobiotechnology. 2026 Feb 3;24:130. doi: 10.1186/s12951-026-04080-3 (PMC12879337; doi:10.1186/s12951-026-04080-3)
Supplement: Supplementary file 1 — Supplementary Material 1 [file 12951_2026_4080_MOESM1_ESM.docx]

**Supporting Information**

**AE-MXene-Modified Titanium Alloy Promotes Osseointegration by Regulating the AMPK-MTOR-Autophagy Pathway in Macrophage**

Rui Chao ^a, 1^, Lei Sun ^b, 1^, Xinyu Xu ^c, 1^, Zhan Liu ^a^, Xinyi Xu ^b^, Zhen Ren ^c^, Xinwei Chen ^a^, Weifeng Xu ^a^, Xuzhuo Chen ^a, *^, Ying Hu ^c, *^, Shanyong Zhang ^a, *^

*^a^ Department of Oral Surgery, Shanghai Ninth People’s Hospital, Shanghai Jiao Tong University School of Medicine; College of Stomatology, Shanghai Jiao Tong University; National Center for Stomatology; National Clinical Research Center for Oral Diseases; Shanghai Key Laboratory of Stomatology; Shanghai Research Institute of Stomatology, Shanghai, 200011, China.*

*^b^ Hefei Stomatology Hospital and Hefei Clinical School of Stomatology, Anhui Medical University. Hefei 230001, Anhui Province, China.*

*^c^ Anhui Province Key Lab of Aerospace Structural Parts Forming Technology and Equipment, School of Materials Science and Engineering, Hefei University of Technology, Hefei, 230009, China*

^*^ Corresponding authors: E-mail addresses: cxzzxcaa@126.com (X. Chen); huying@hfut.edu.cn (Y. Hu); zhangsy1787@sh9hospital.org.cn (S. Zhang).

^1^ Rui Chao and Lei Sun contributed equally to this work.


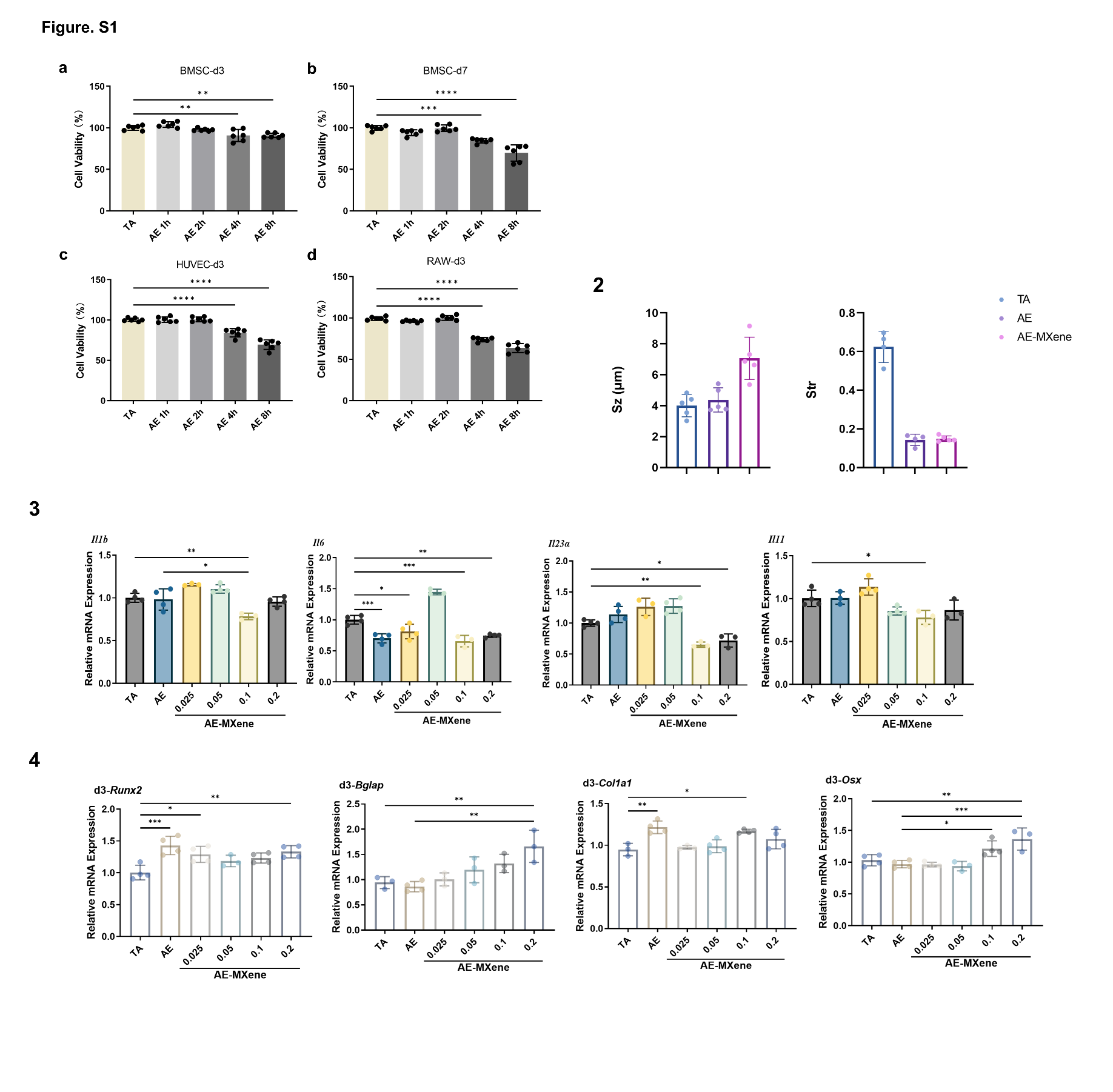


**Figure S1.** CCK-8 assays of different alkali etching times in BMSCs (a, b), HUVECs (c), and RAW264.7 cells (d). n ≥ 3. ***P*<0.01, ****P*<0.001, and *****P*<0.0001.


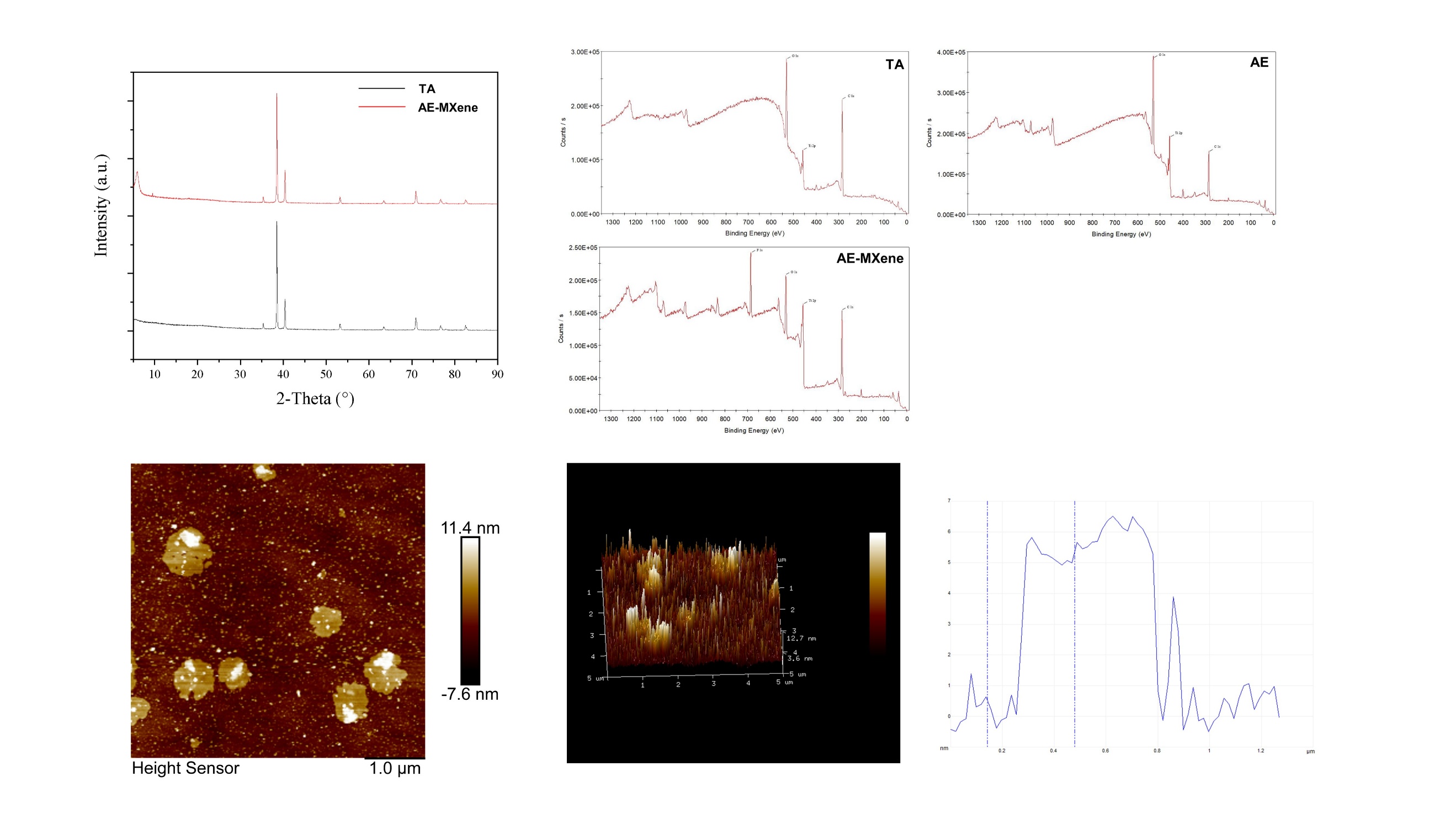


**Figure S2.** XPS analysis of TA, AE and AE-MXene (0.1 mg/ml).


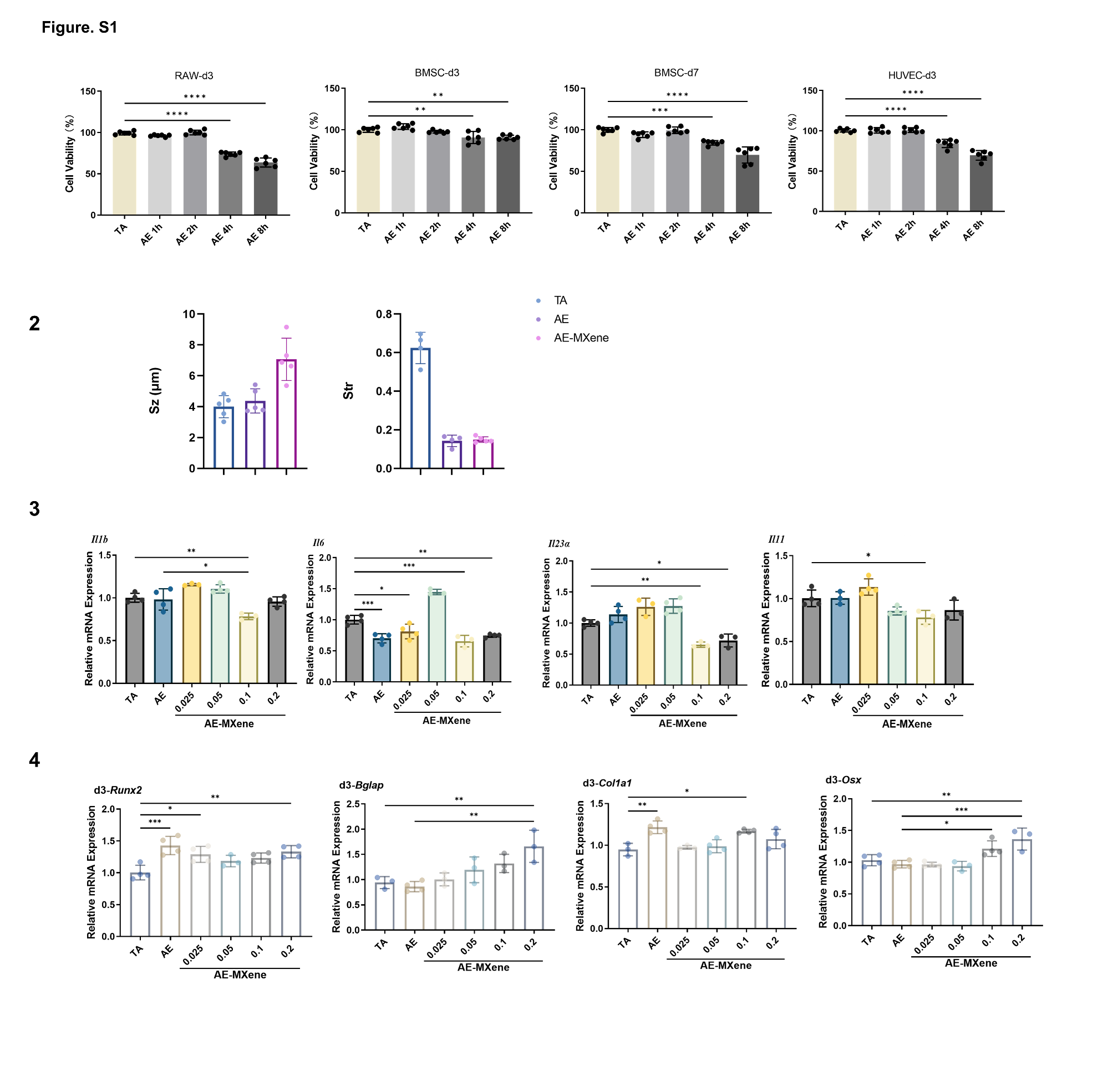


**Figure S3.** Statistical plots of surface roughness for Sz and Str. n ≥ 3.


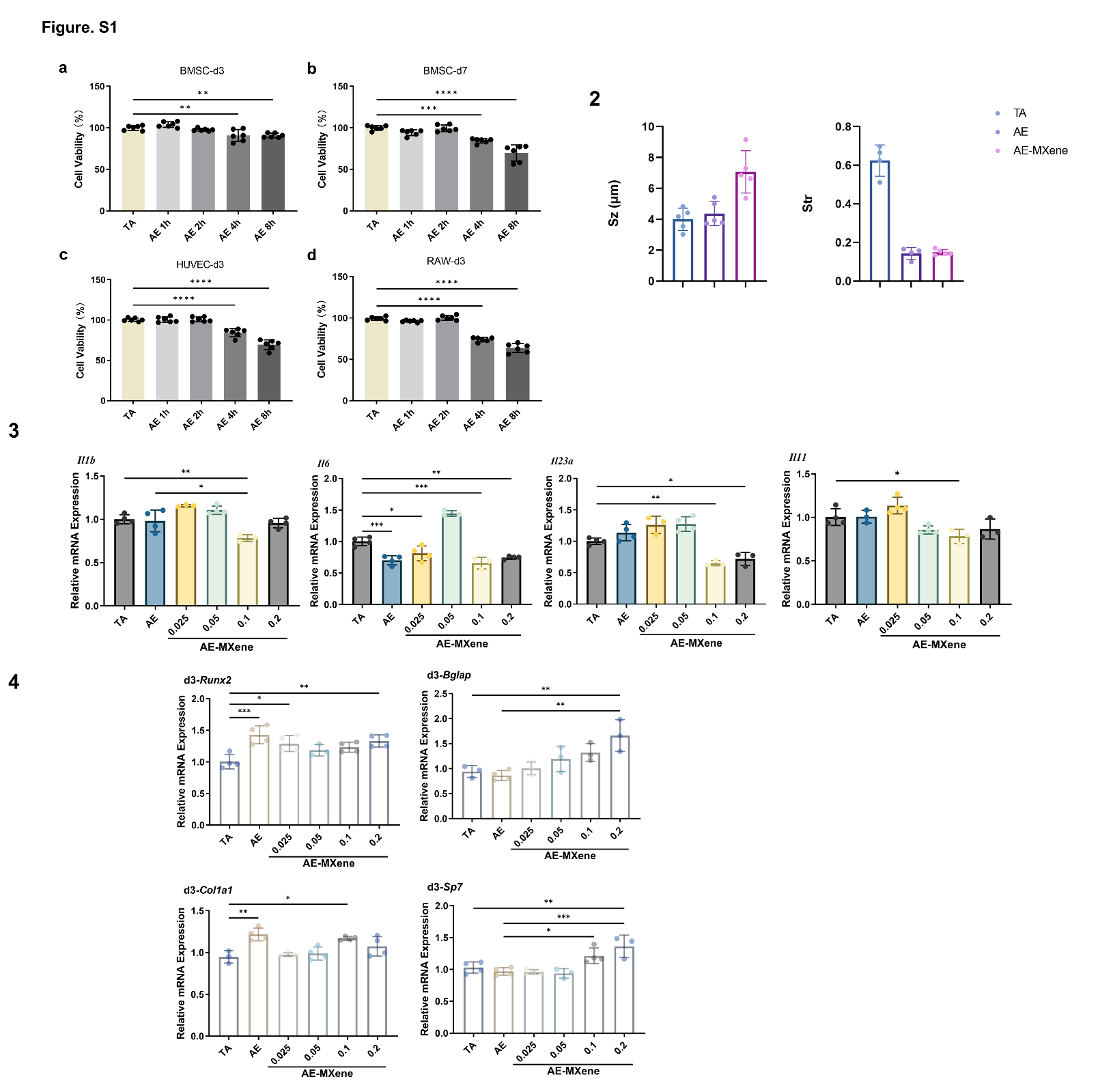


**Figure S4.** Expression of inflammatory factor-related genes analyzed by RT-qPCR in macrophages in the different groups. The unit of AE-MXene was mg/ml. n ≥ 3. **P*<0.05, ***P*<0.01, ****P*<0.001.


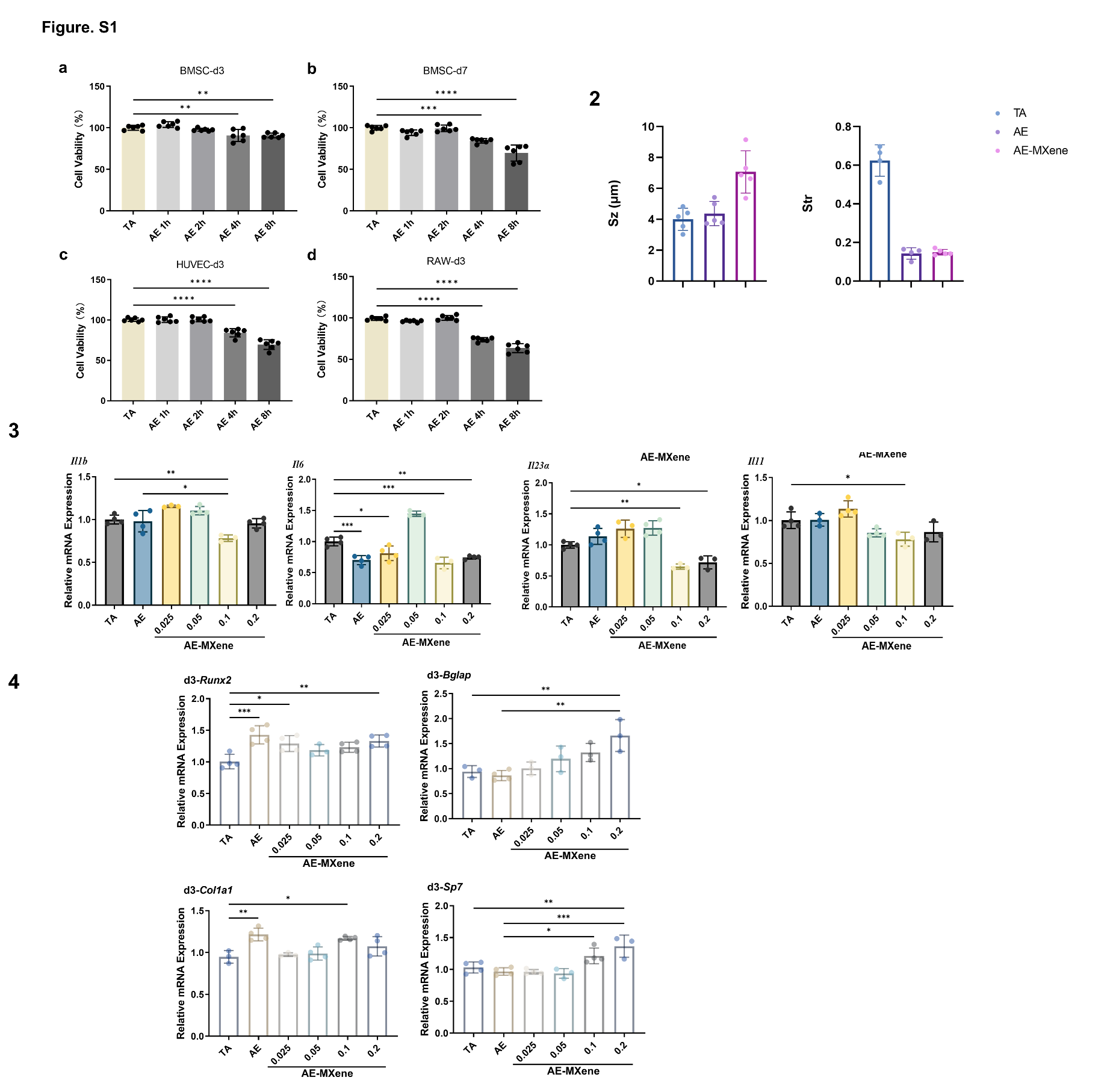


**Figure S5.** Expression of osteogenesis-related genes by RT-qPCR in BMSCs in the different groups with the conditional culture on the third day. The unit of AE-MXene was mg/ml. n ≥ 3. **P*<0.05, ***P*<0.01, ****P*<0.001.


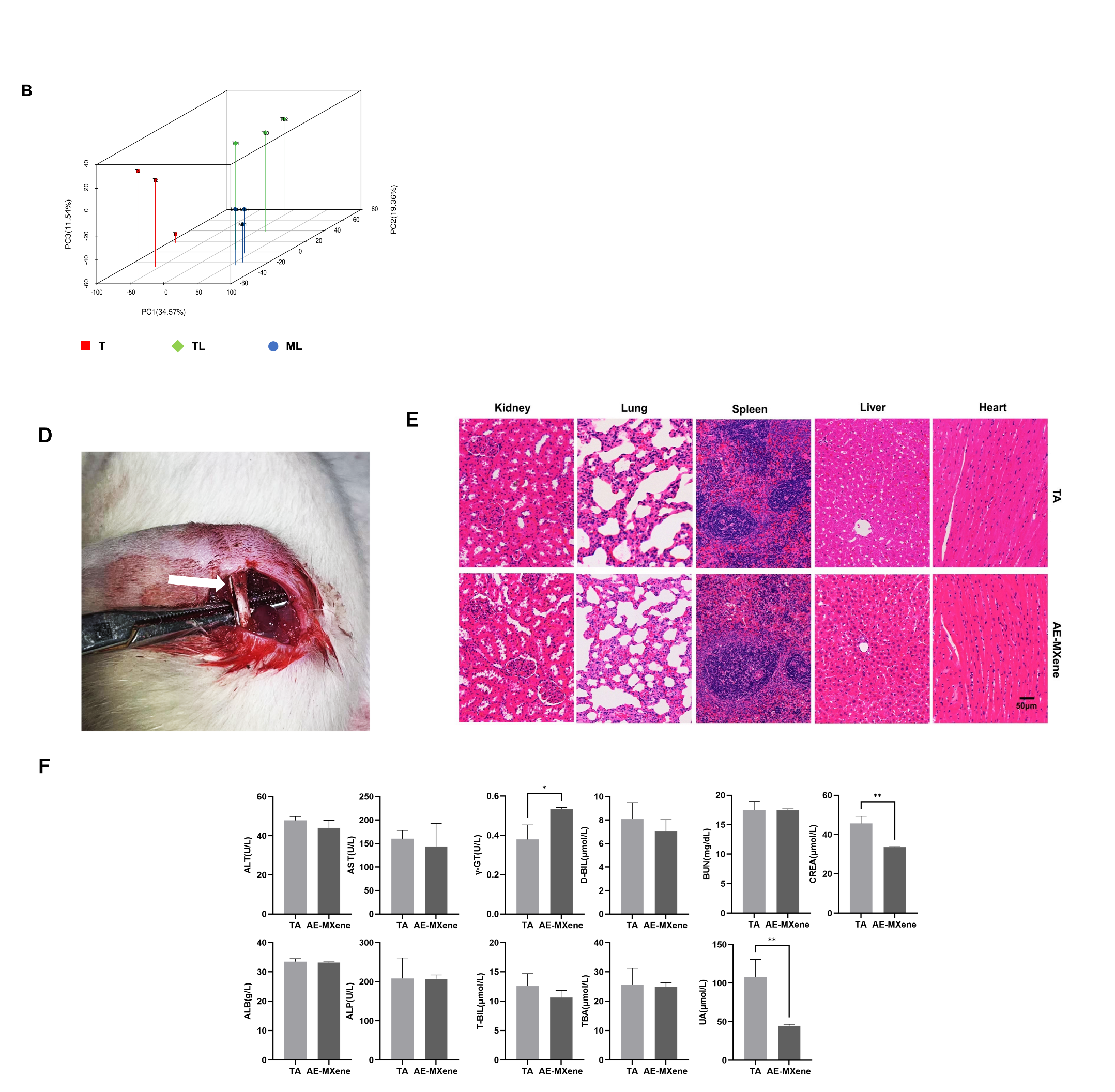


**Figure S6.** PCA plot of the T (TA), TL (TA+LPS) and ML (AE-MXene (0.1mg/ml) +LPS) experimental groups. n ≥ 3.


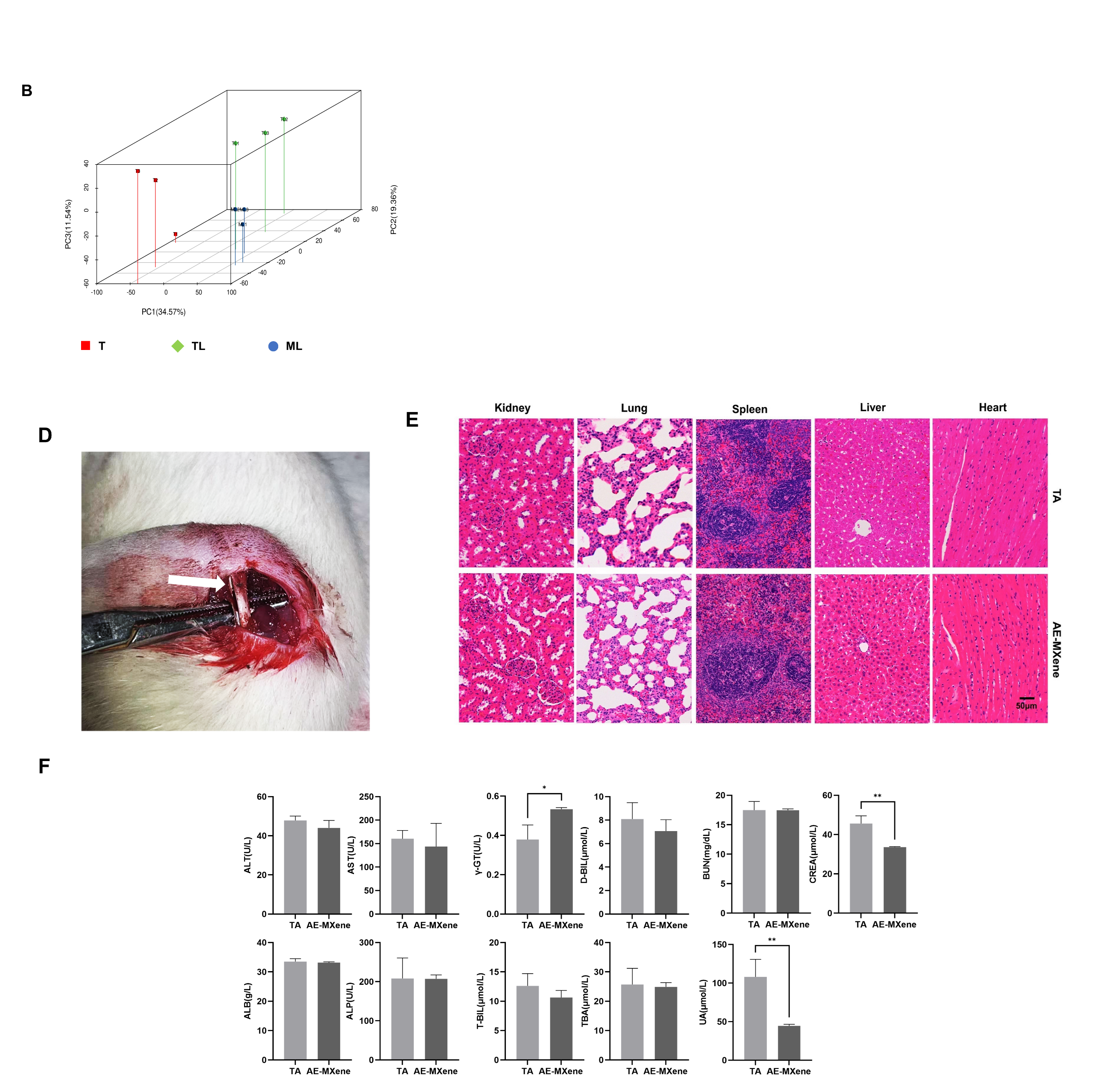


**Figure S7.** Representative H&E-stained images of primary organ tissues (kidney, lung, spleen, liver, and heart) of experimental rats 4 weeks after implantation. n ≥ 3. Scale bar = 50 μm.


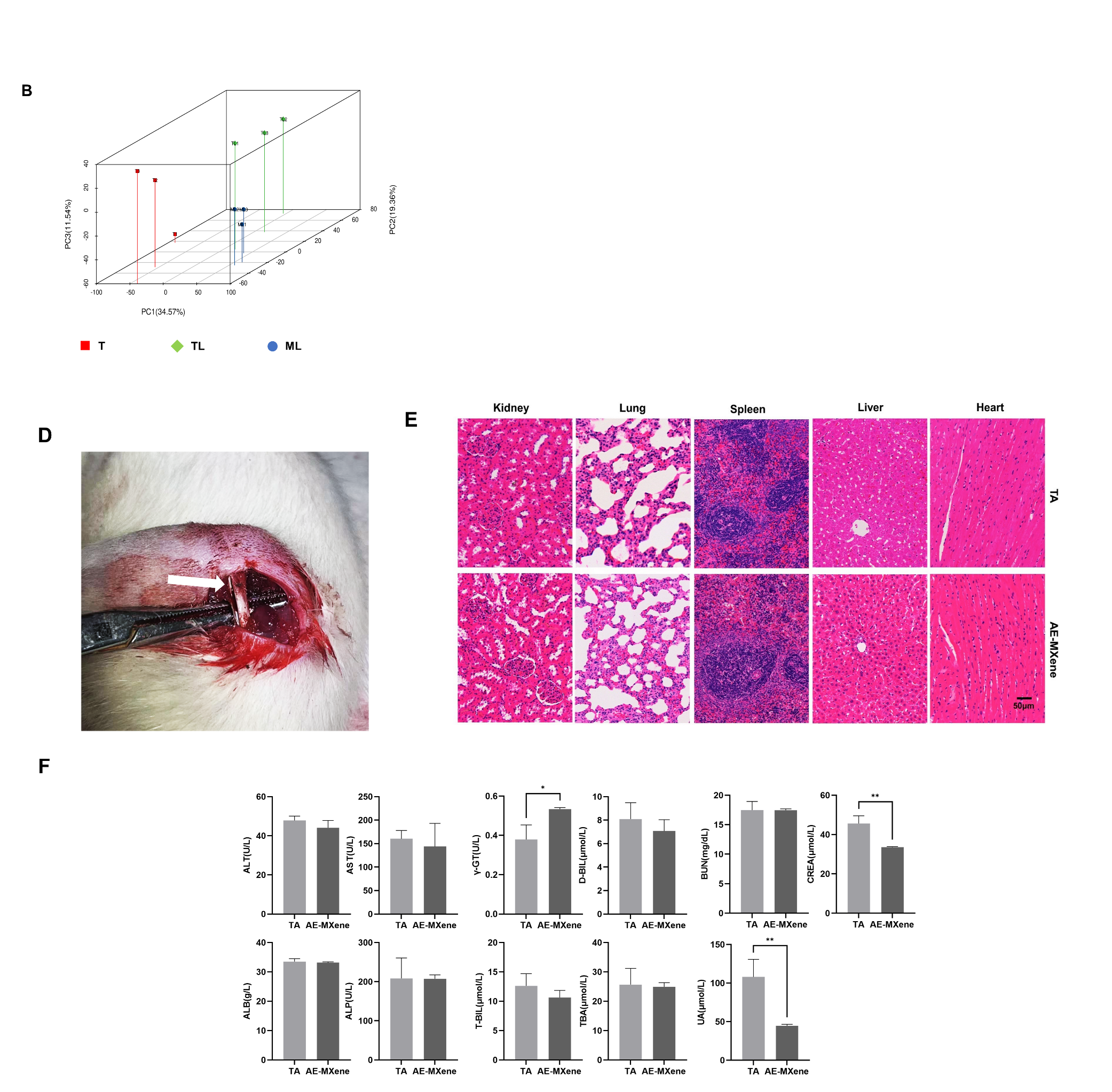


**Figure S8.** The heart blood of rats in different material groups was collected after deep anesthesia. Serum samples were taken after heart for liver and renal function tests. n ≥ 3. **P*<0.05, ***P*<0.01.


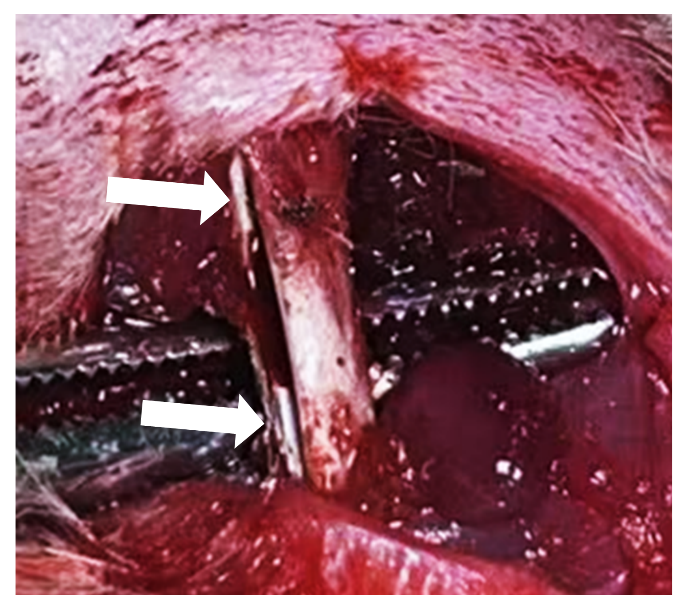


**Figure S9.** Immediate intraoperative image of successful implantation of the material in the rat. The white arrow marks the implanted titanium disc.


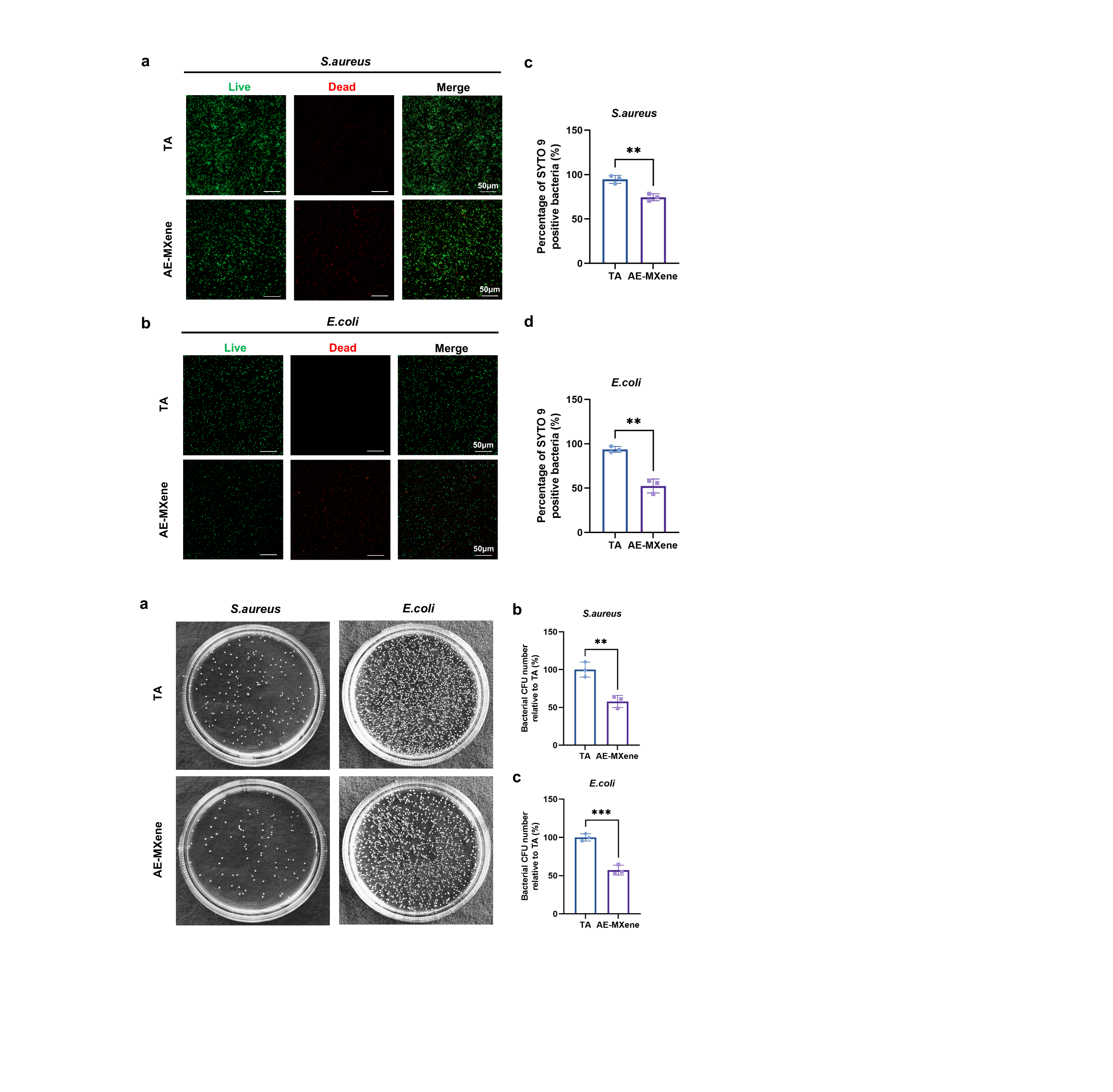


**Figure S10.** The Live/Dead staining images of (a) *S. aureus* and (b) *E. coli* cultured on TA and AE-MXene surfaces. (c, d) Quantitative analysis of the percentage of SYTO 9 positive bacteria relative to TA. n ≥ 3. Scale bar = 50 μm. ***P*<0.01.


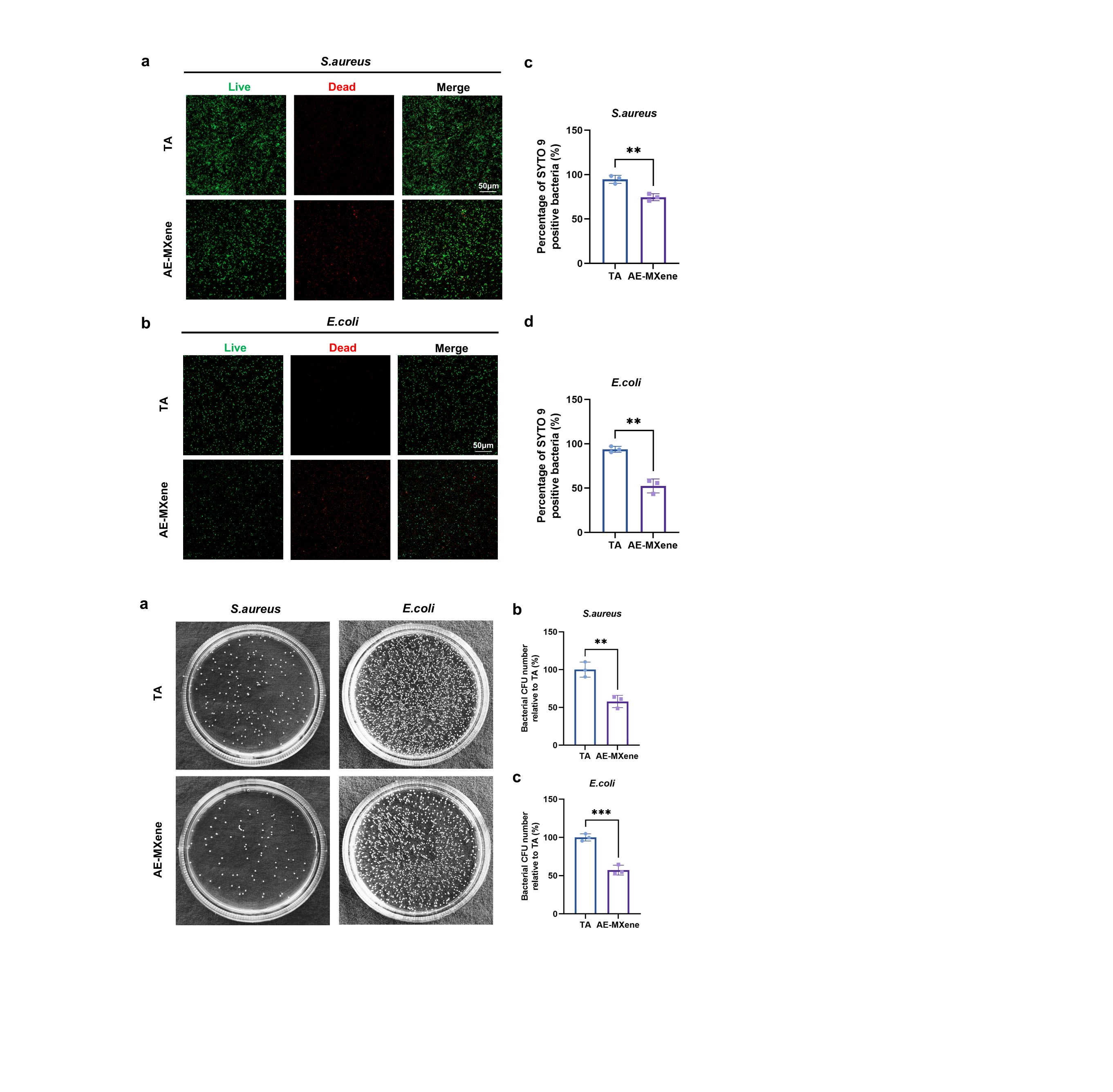


**Figure S11.** (a) Representative images of *S. aureus* and *E. coli* grown on the surface of TA and AE-MXene. (b) Quantitative analysis of *S. aureus* CFU number relative to TA. (c) Quantitative analysis of *E. coli* CFU number relative to TA. n ≥ 3. ***P*<0.01, ****P*<0.001.


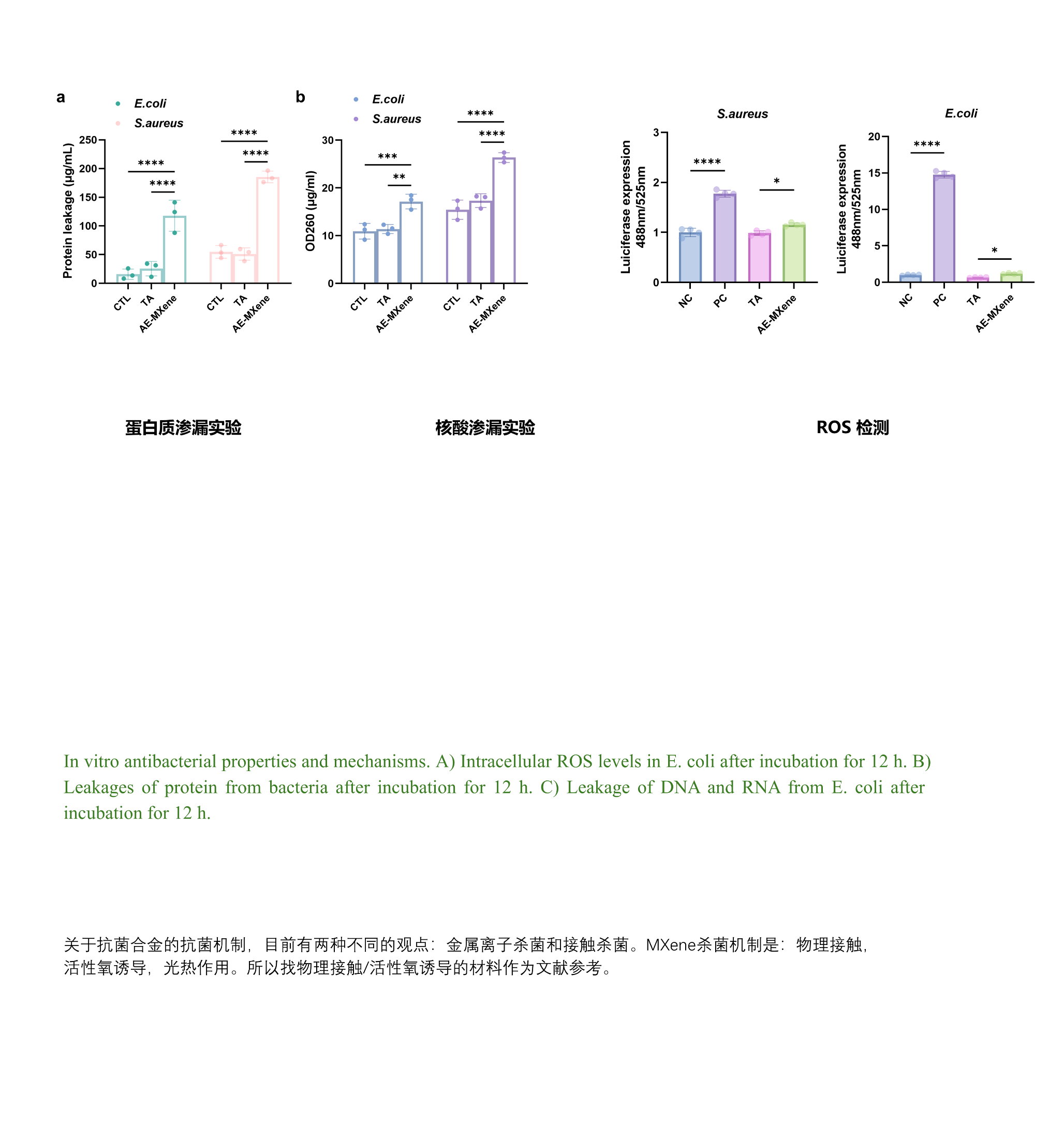


**Figure S12.** Quantitative images of reactive oxygen species (ROS) detection in *S. aureus* and *E. coli* cultured on different material surfaces. Experimental groups: NC, negative control (bacteria without treatment or dye); PC, positive control (bacteria with ROS-sensitive dye); TA, bacteria cultured on titanium alloy (TA) surfaces; AE-MXene, bacteria cultured on alkali-etched MXene (AE-MXene) surfaces. ROS production was assessed using a fluorescent dye, with signal intensity proportional to ROS levels. n ≥ 3. **P*<0.05, *****P*<0.0001.


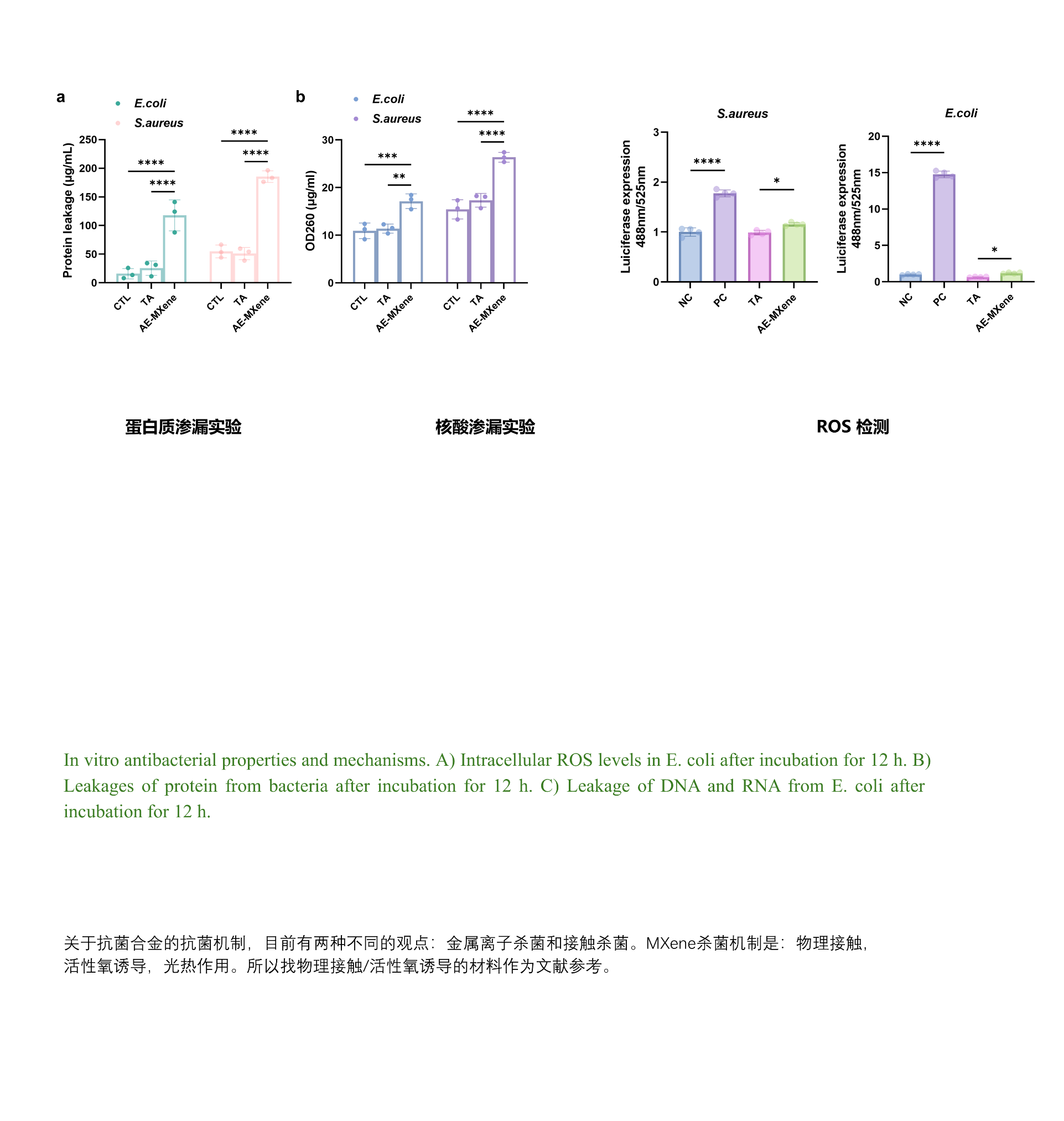


**Figure S13.** Quantitative assessment of protein and nucleic acid leakage from *S. aureus* and *E. coli* cultured on different material surfaces. *S. aureus* and *E. coli* were respectively cultured on the surfaces of TA material and AE-MXene material for 12 hours. After co-culture, the bacterial supernatants were collected for analysis. (a) Concentrations of leaked proteins from *S. aureus* and *E. coli*. (b) Concentrations of leaked nucleic acids (at 260 nm) from *S. aureus* and *E. coli*. n ≥ 3. ***P*<0.01, ****P*<0.001, *****P*<0.0001.


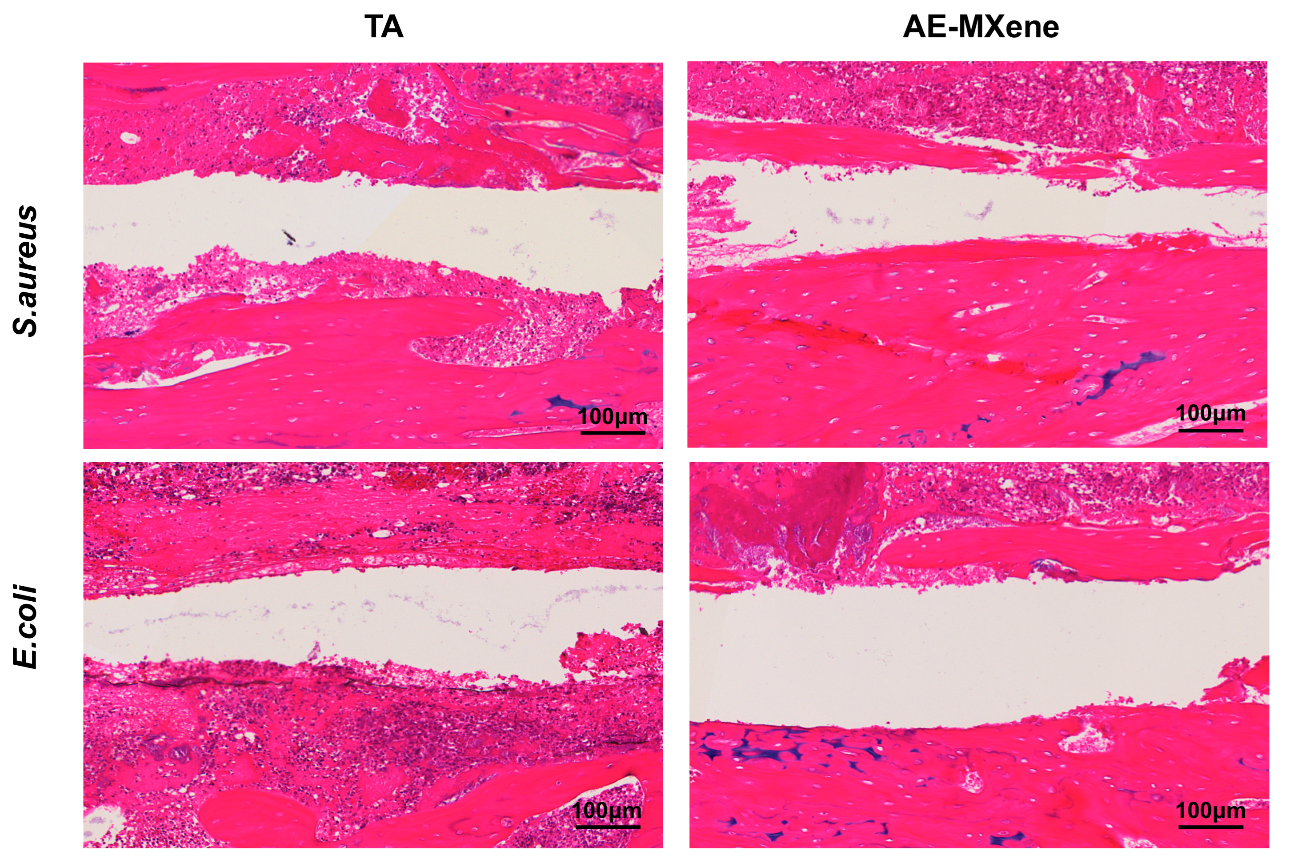


**Figure S14.** H&E staining images of rat femurs implanted with different material groups (immersed in *S. aureus* and *E. coli* suspension, respectively). Scale bar = 100 μm.


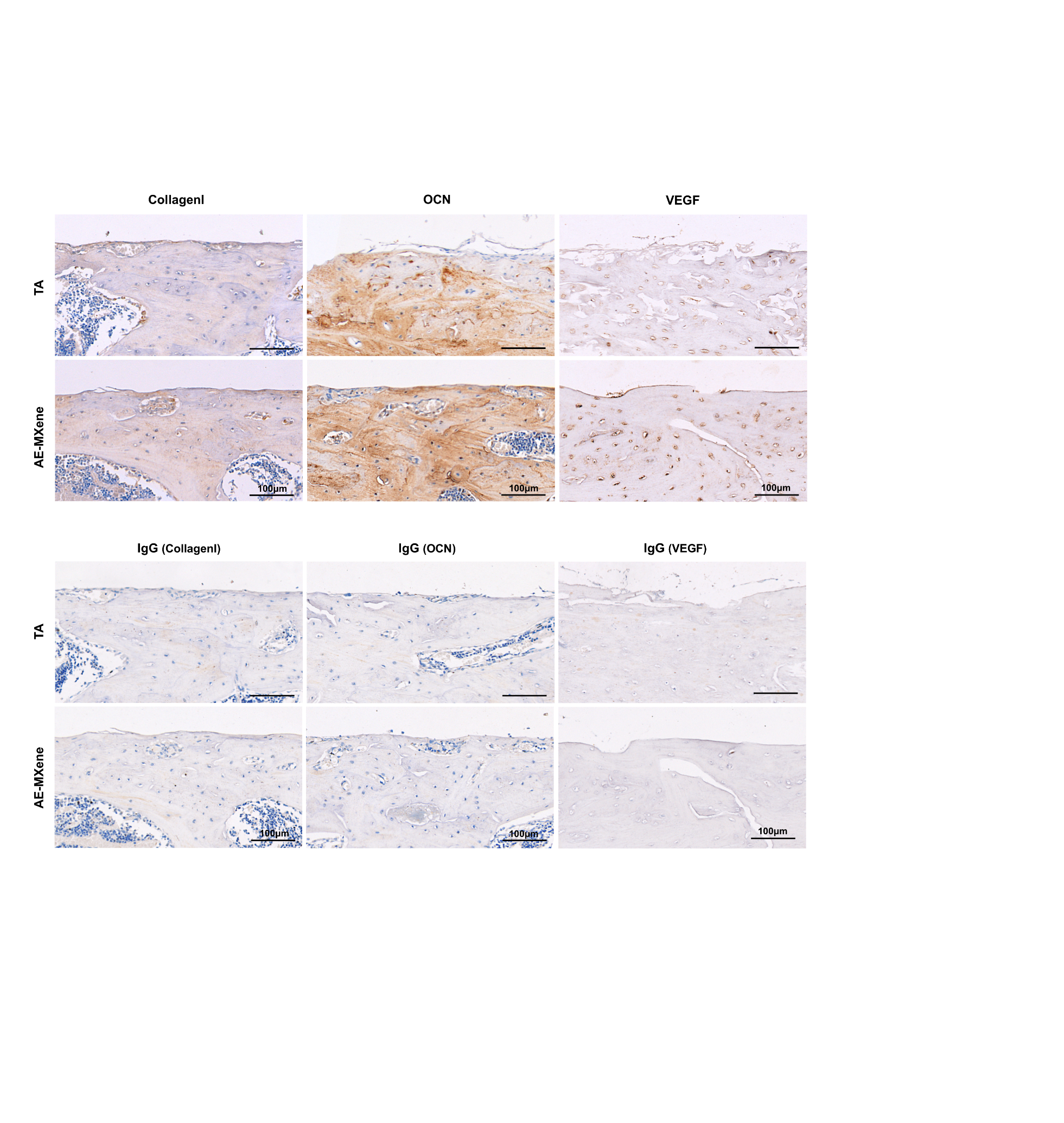


**Figure S15.** Specificity of immunostaining for Collagen I, osteocalcin (OCN), and vascular endothelial growth factor (VEGF) was verified via negative control experiments. Negative controls were performed by replacing primary antibodies with non-immune IgG in TA and AE-MXene samples following titanium rod removal. Scale bar = 100 μm.

**Table 1. Primers used in Quantitative Real-Time PCR.**

| Name | Primer sequence |
| --- | --- |
| *Gapdh* | Forward: 5ʹ-AGGTCGGTGTGAACGGATTTG-3ʹ  Reverse: 5ʹ-TGTAGACCATGTAGTTGAGGTCA-3ʹ |
| *GAPDH* | Forward: 5ʹ-GGAGCGAGATCCCTCCAAAAT-3ʹ  Reverse: 5ʹ-GGCTGTTGTCATACTTCTCATGG-3ʹ |
| *Il1b* | Forward: 5ʹ-TGAGCACCTTCTTTTCCTTCATC-3ʹ  Reverse: 5ʹ-TGTCTAATGGGAACGTCACACAC-3ʹ |
| *Il6* | Forward: 5ʹ-GAGCCCACCAAGAACGATAG-3ʹ  Reverse: 5ʹ-TTTCCACGATTTCCCAGAGA-3ʹ |
| *Il11* | Forward: 5ʹ-TGTTCTCCTAACCCGATCCCT-3ʹ  Reverse: 5ʹ-CAGGAAGCTGCAAAGATCCCA-3ʹ |
| *Il18* | Forward: 5ʹ-GACTCTTGCGTCAACTTCAAGG-3ʹ  Reverse: 5ʹ-CAGGCTGTCTTTTGTCAACGA-3ʹ |
| *Il23a* | Forward: 5ʹ-ATGCTGGATTGCAGAGCAGTA-3ʹ  Reverse: 5ʹ-ACGGGGCACATTATTTTTAGTCT-3ʹ |
| *Mrc1* | Forward: 5ʹ-CTCTGTTCAGCTATTGGACGC-3ʹ  Reverse: 5ʹ-CGGAATTTCTGGGATTCAGCTTC-3ʹ |
| *Arg1* | Forward: 5ʹ-CTCCAAGCCAAAGTCCTTAGAG-3ʹ  Reverse: 5ʹ-AGGAGCTGTCATTAGGGACATC-3ʹ |
| *Pparg* | Forward: 5ʹ-TCGCTGATGCACTGCCTATG-3ʹ  Reverse: 5ʹ-GAGAGGTCCACAGAGCTGATT-3ʹ |
| *Retnla* | Forward: 5ʹ-CCAATCCAGCTAACTATCCCTCC-3ʹ  Reverse: 5ʹ-ACCCAGTAGCAGTCATCCCA-3ʹ |
| *Runx2* | Forward: 5ʹ-ATGCTTCATTCGCCTCACAAA-3ʹ  Reverse: 5ʹ-GCACTCACTGACTCGGTTGG-3ʹ |
| *Bglap* | Forward: 5ʹ-GCAATAAGGTAGTGAACAGACTCC-3ʹ  Reverse: 5ʹ-CCATAGATGCGTTTGTAGGCGG-3ʹ |
| *Col1a1* | Forward: 5ʹ-GCTCCTCTTAGGGGCCACT-3ʹ  Reverse: 5ʹ-CCACGTCTCACCATTGGGG-3ʹ |
| *Sp7* | Forward: 5ʹ-ATGGCGTCCTCTCTGCTTG-3ʹ  Reverse: 5ʹ-TGAAAGGTCAGCGTATGGCTT-3ʹ |
| *Alpl* | Forward: 5ʹ-CCAACTCTTTTGTGCCAGAGA-3ʹ  Reverse: 5ʹ-GGCTACATTGGTGTTGAGCTTTT-3ʹ |
| *Spp1* | Forward: 5ʹ-AGCAAGAAACTCTTCCAAGCAA-3ʹ  Reverse: 5ʹ-GTGAGATTCGTCAGATTCATCCG-3ʹ |
| *VEGF* | Forward: 5ʹ-AGGGCAGAATCATCACGAAGT-3ʹ  Reverse: 5ʹ-AGGGTCTCGATTGGATGGCA-3ʹ |
| *ANG* | Forward: 5ʹ-CTGGGCGTTTTGTTGTTGGTC-3ʹ  Reverse: 5ʹ-GGTTTGGCATCATAGTGCTGG-3ʹ |
| *Ulk1* | Forward: 5ʹ-AAGTTCGAGTTCTCTCGCAAG-3ʹ  Reverse: 5ʹ-CGATGTTTTCGTGCTTTAGTTCC-3ʹ |
| *Sqstm1* | Forward: 5ʹ-AGGATGGGGACTTGGTTGC-3ʹ  Reverse: 5ʹ-TCACAGATCACATTGGGGTGC-3ʹ |
| *Becn1* | Forward: 5ʹ-ATGGAGGGGTCTAAGGCGTC-3ʹ  Reverse: 5ʹ-TCCTCTCCTGAGTTAGCCTCT-3ʹ |
| *Atg5* | Forward: 5ʹ-TGTGCTTCGAGATGTGTGGTT-3ʹ  Reverse: 5ʹ-GTCAAATAGCTGACTCTTGGCAA-3ʹ |
| *Map1lc3* | Forward: 5ʹ-GACCGCTGTAAGGAGGTGC-3ʹ  Reverse: 5ʹ-CTTGACCAACTCGCTCATGTTA-3ʹ |
