# Supplementary figures and images for: AE-MXene-modified titanium alloy promotes osseointegration by regulating the AMPK-MTOR-autophagy pathway in macrophage
Source: J Nanobiotechnology. 2026 Feb 3;24:130. doi: 10.1186/s12951-026-04080-3 (PMC12879337; doi:10.1186/s12951-026-04080-3)

**Figure 4f**

## Repeat 1

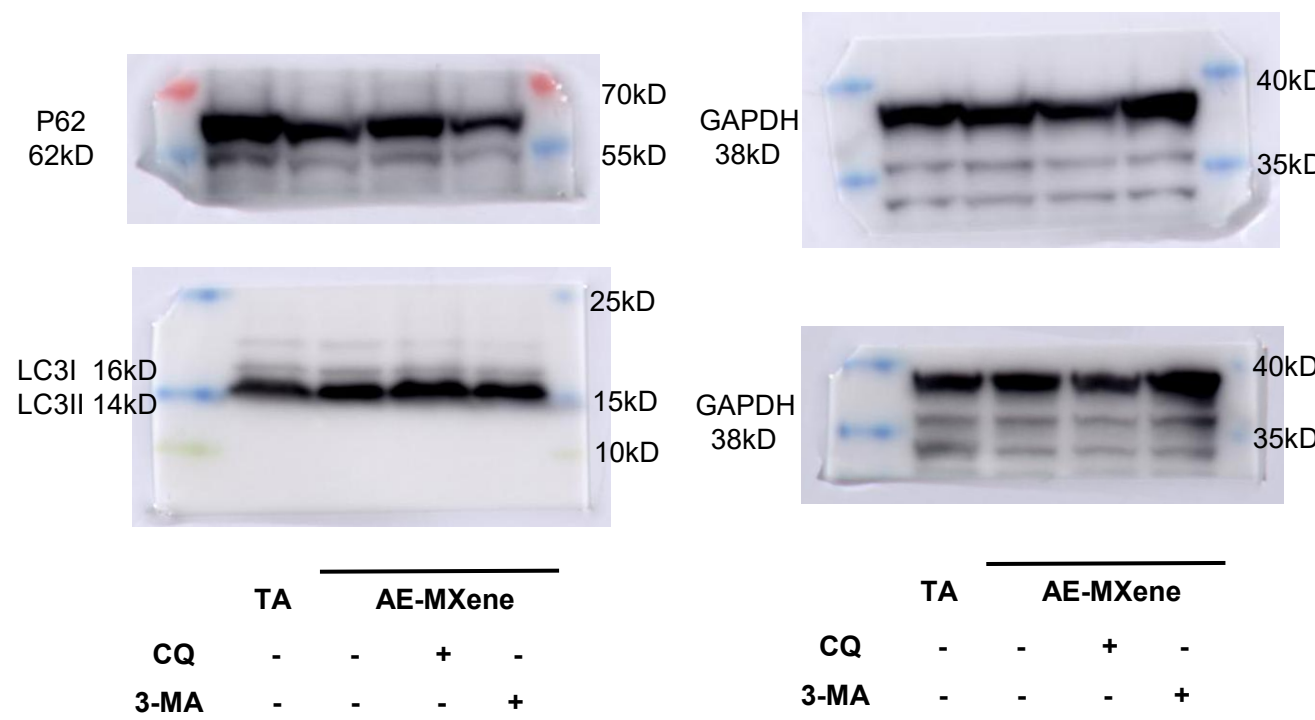

## Repeat 2

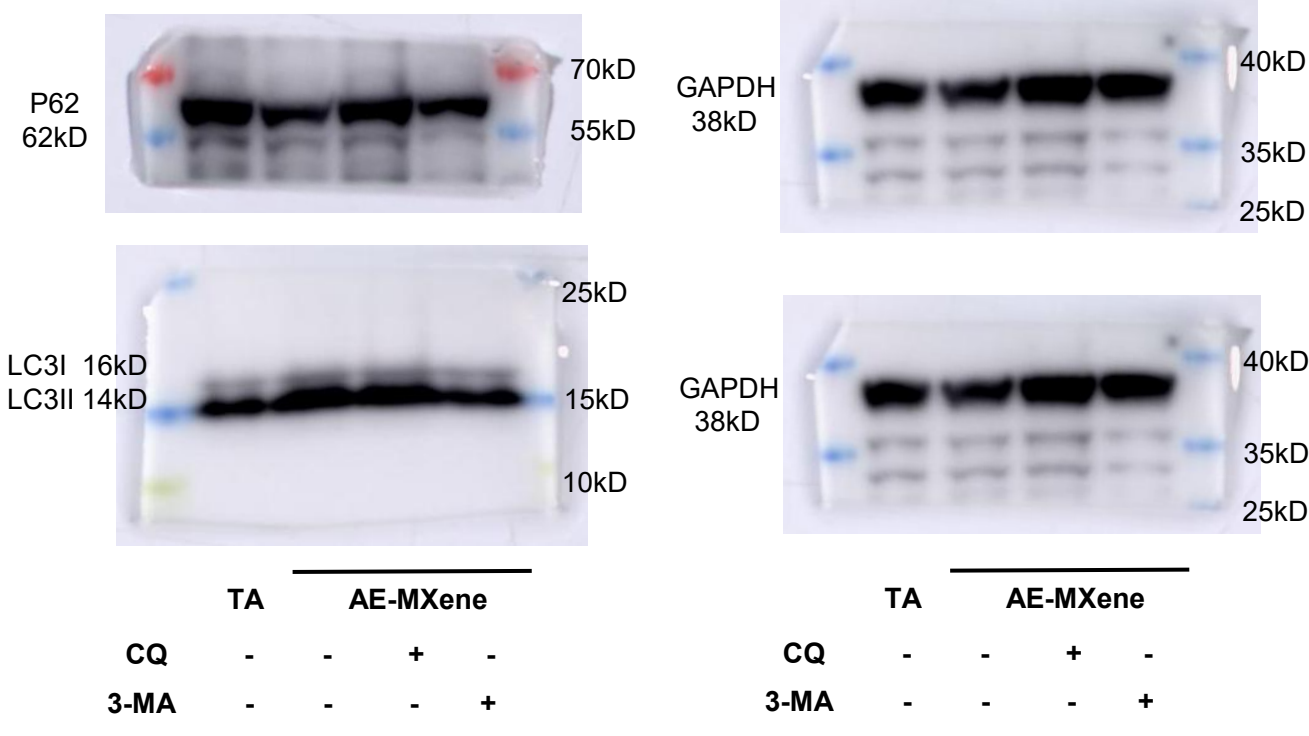

**Repeat 3**

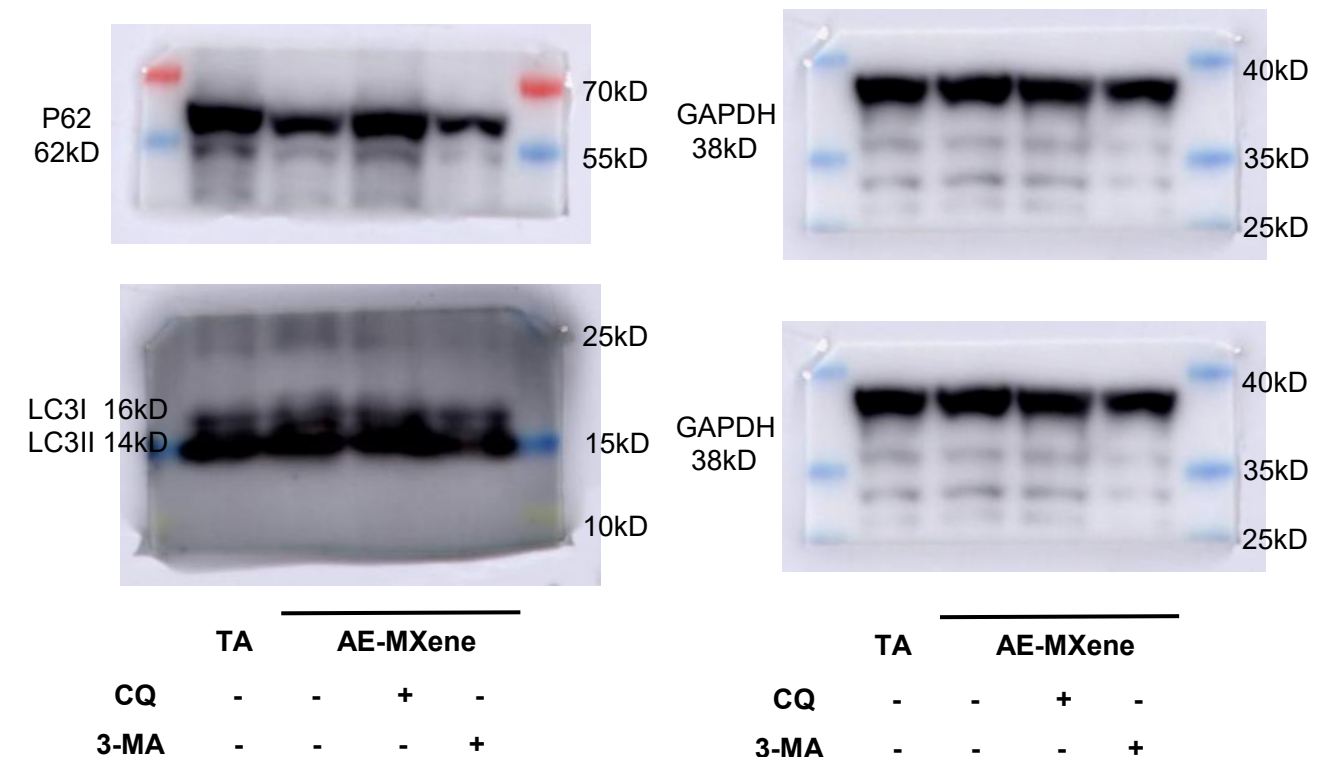

Figure 6a

Repeat 1

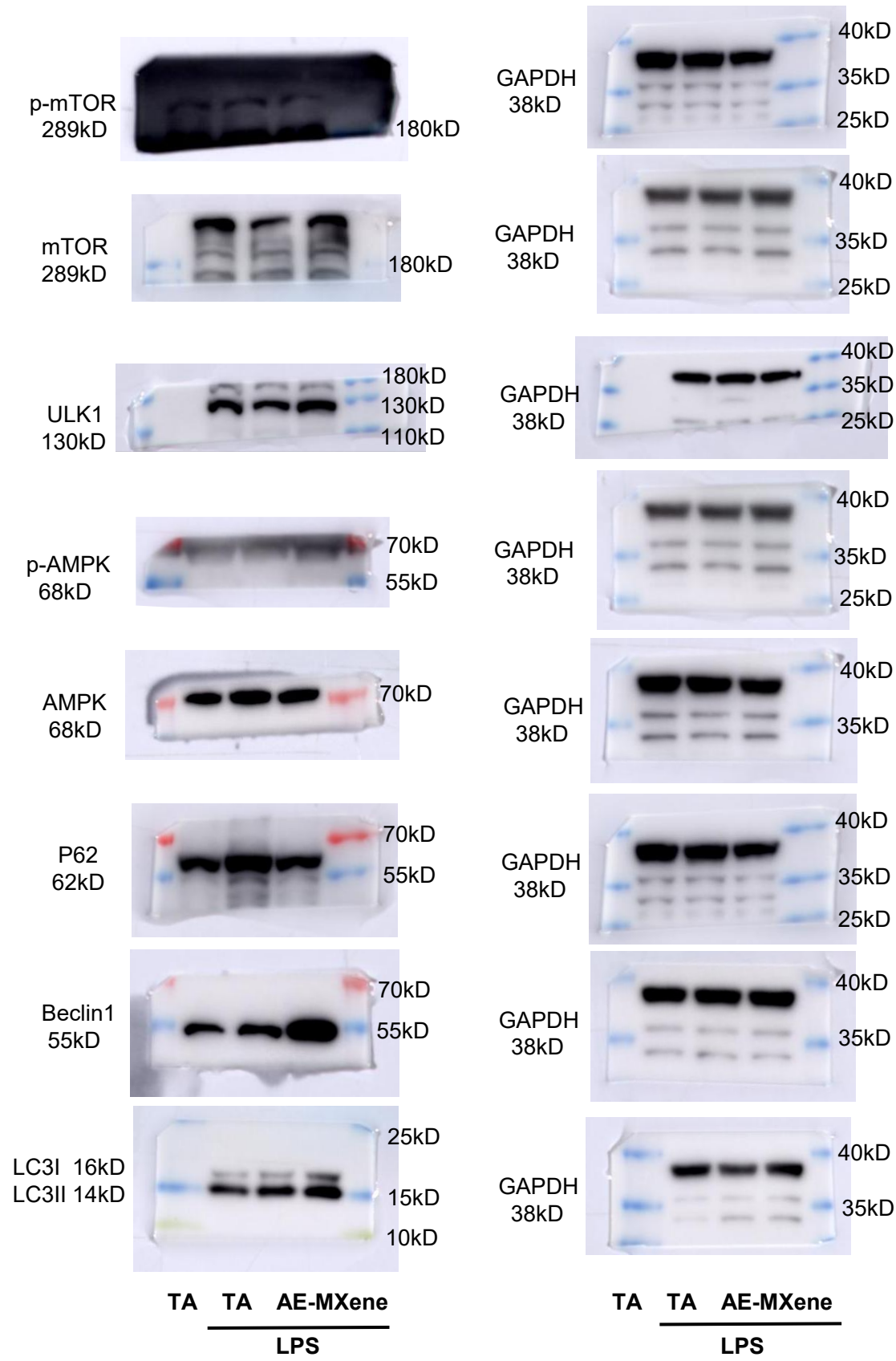

Repeat 2

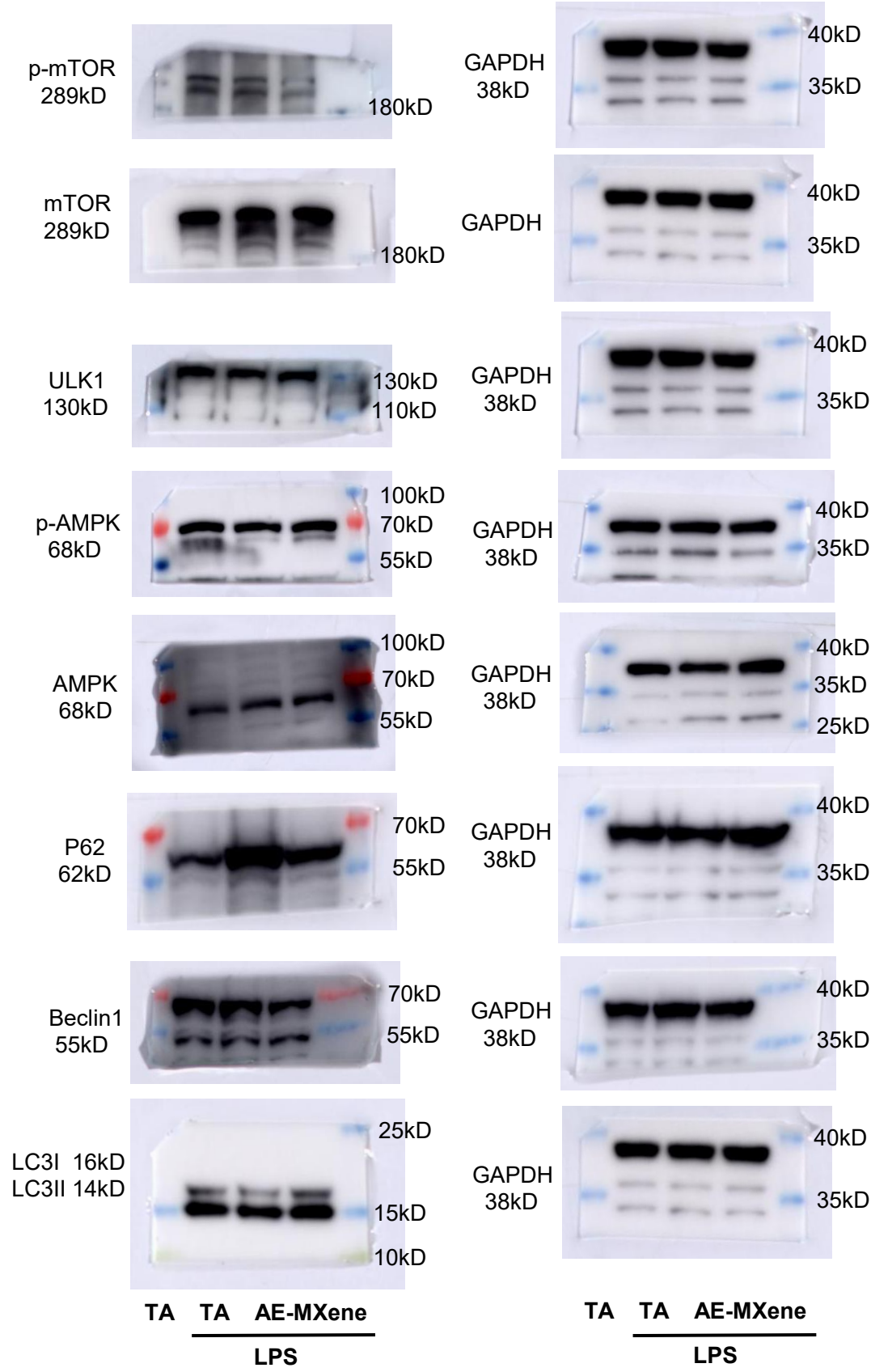

Repeat 3

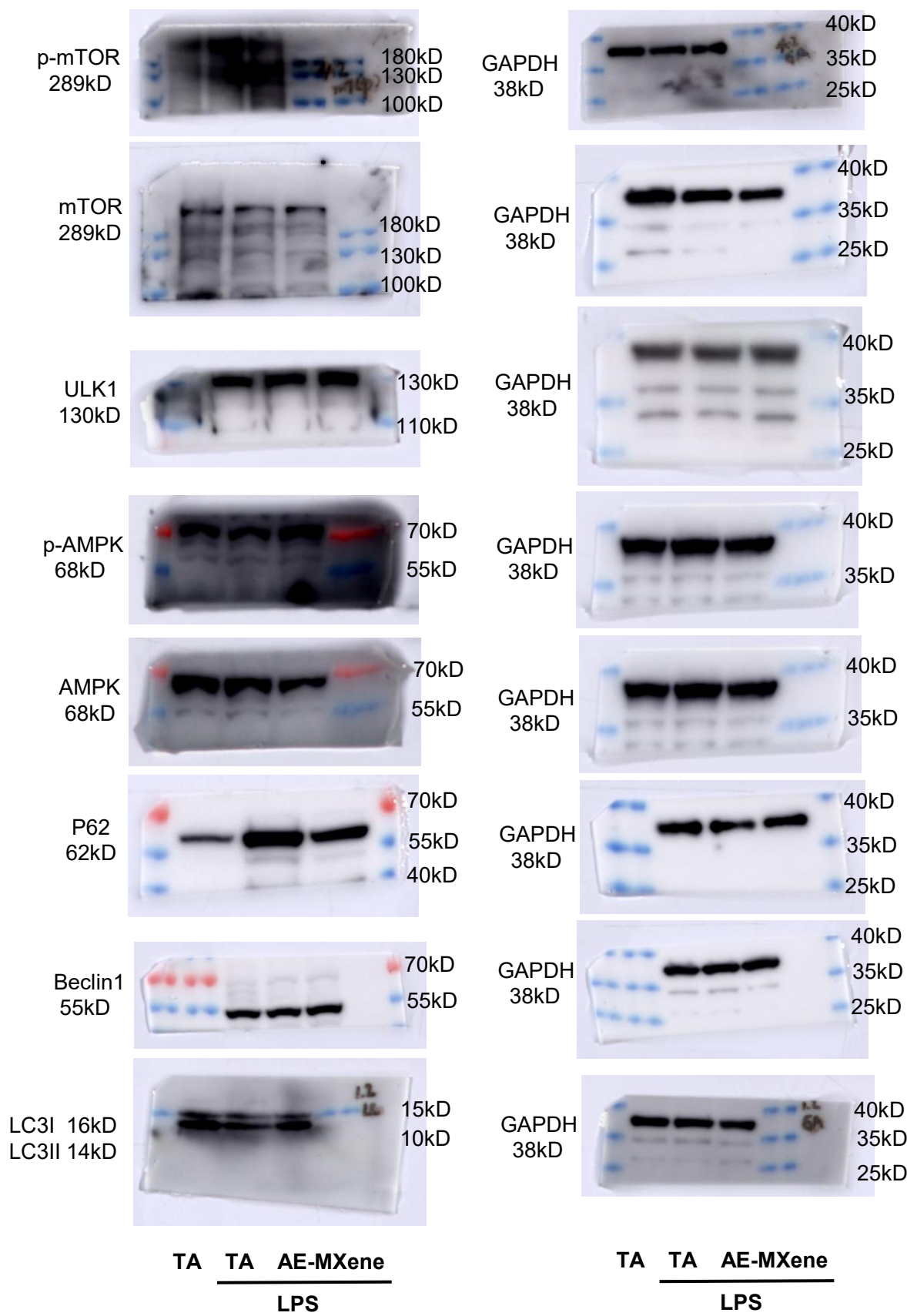

Figure 6e

Repeat 1

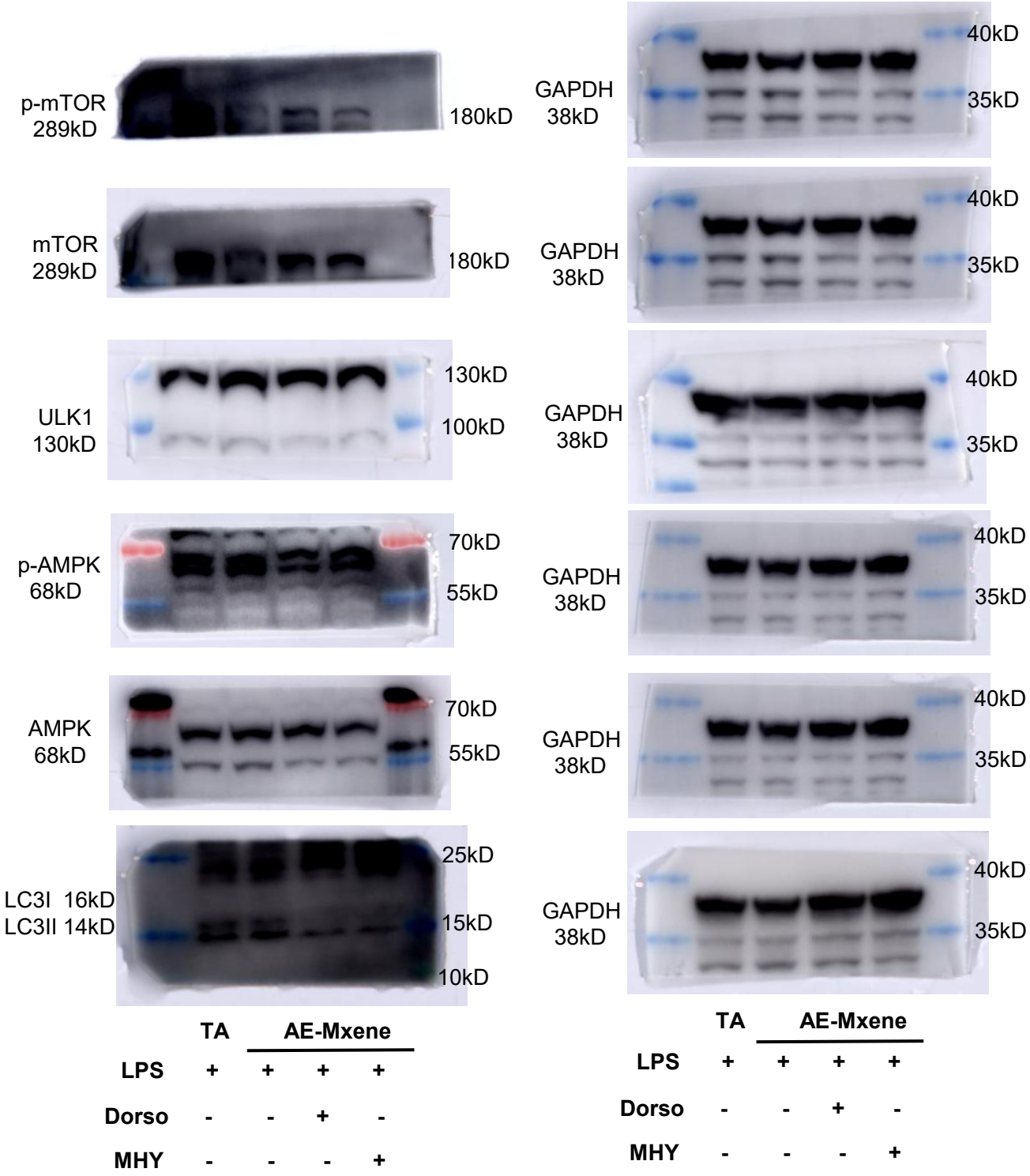

Repeat 2

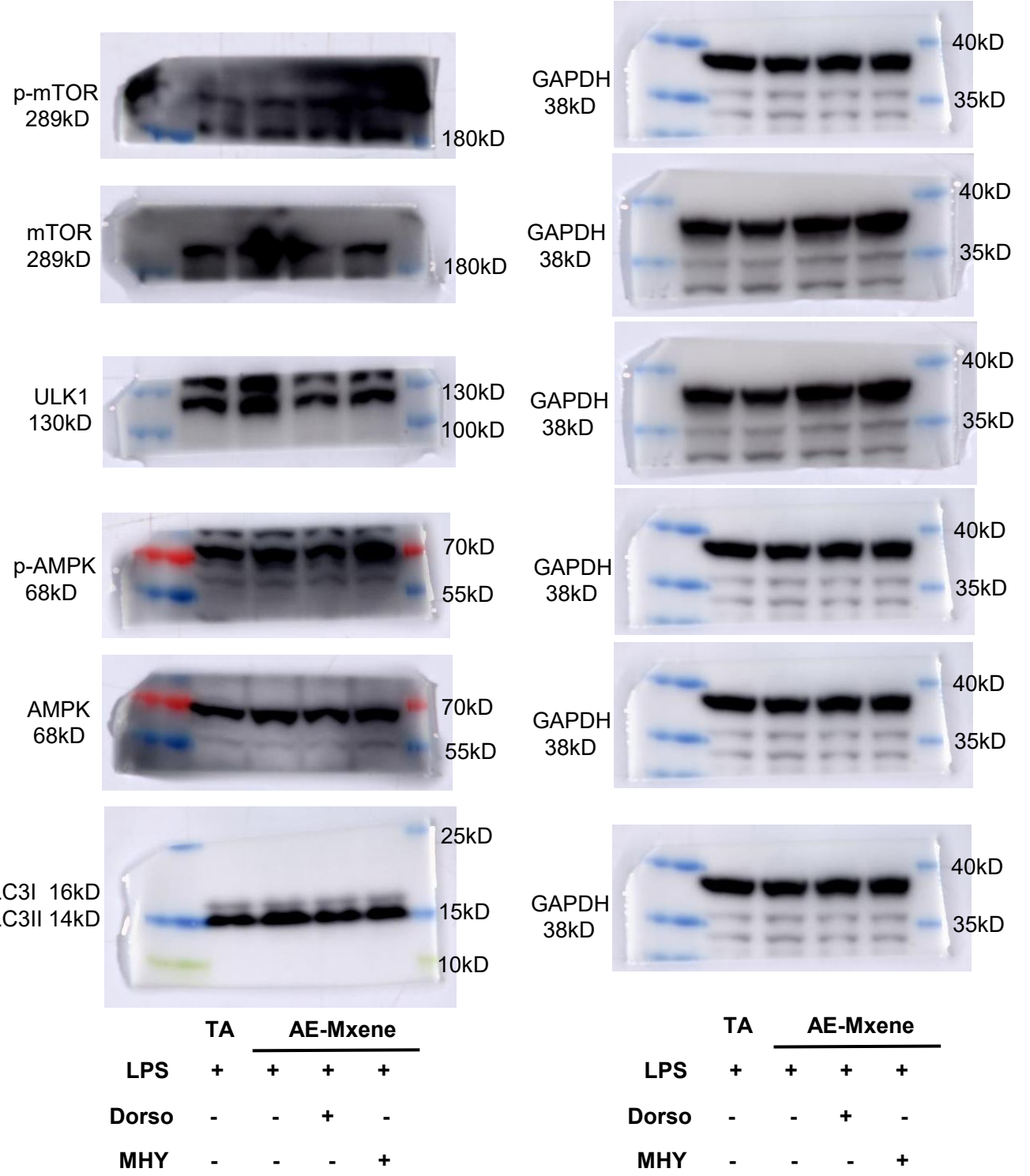

Repeat 3

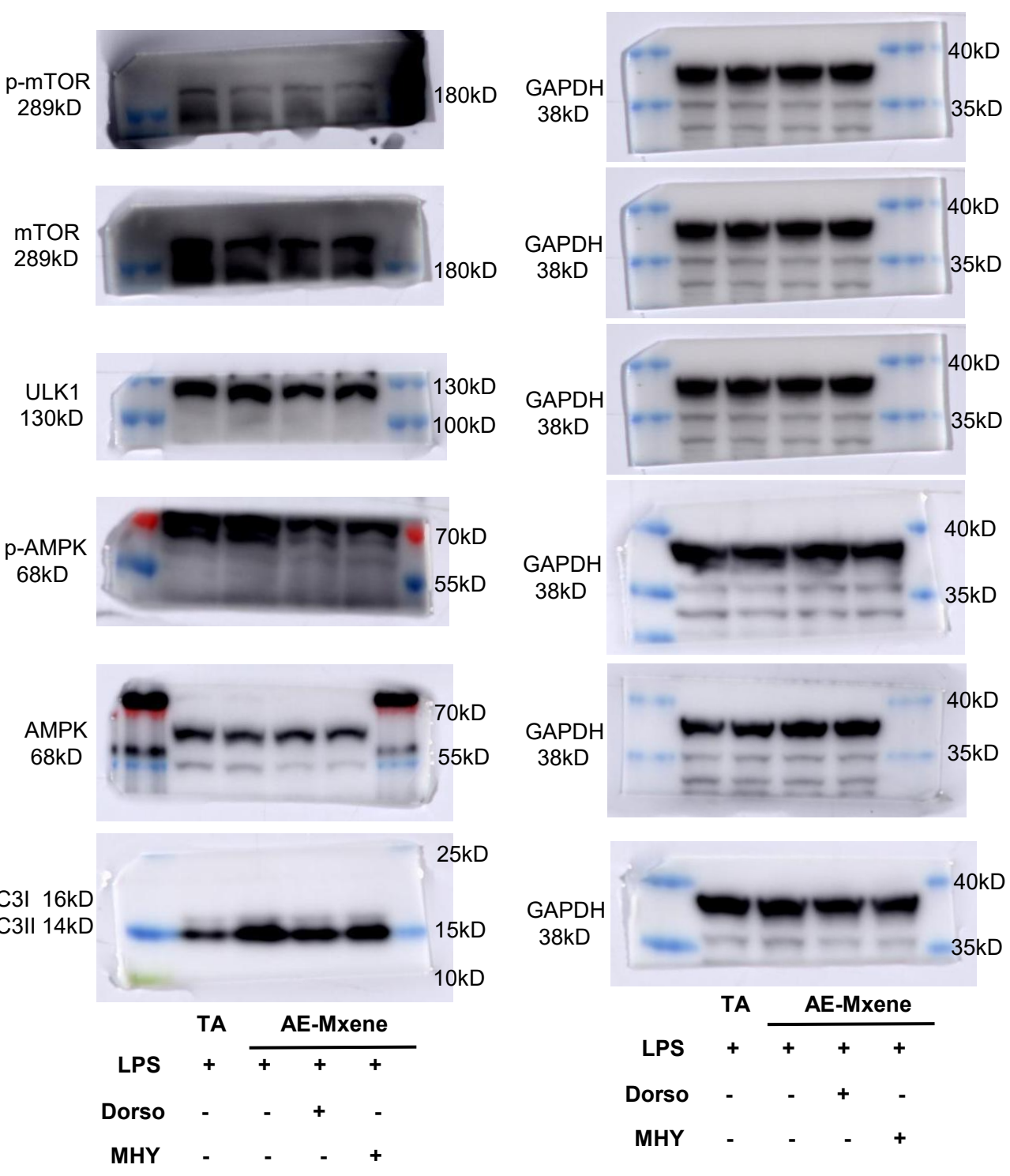

Supplement: Supplementary file 2 — Supplementary Material 2 [file 12951_2026_4080_MOESM2_ESM.pdf]
